# Fused-Ring Oxazolopyrrolopyridopyrimidine Systems with Gram-Negative Activity

**DOI:** 10.3390/antibiotics6010002

**Published:** 2017-01-13

**Authors:** Yiyuan Chen, Jonathan G. Moloney, Kirsten E. Christensen, Mark G. Moloney

**Affiliations:** Chemistry Research Laboratory, Department of Chemistry, University of Oxford, 12 Mansfield Road, Oxford OX1 3TA, UK; cyy412@126.com (Y.C.); jonathan.moloney@stcatz.ox.ac.uk (J.G.M.); kirsten.christensen@chem.ox.ac.uk (K.E.C.)

**Keywords:** antibacterial, tetramate, polyheterocycle, heteroaromatic, annulation

## Abstract

Fused polyheterocyclic derivatives are available by annulation of a tetramate scaffold, and been shown to have antibacterial activity against a Gram-negative, but not a Gram-positive, bacterial strain. While the activity is not potent, these systems are structurally novel showing, in particular, a high level of polarity, and offer potential for the optimization of antibacterial activity.

## 1. Introduction

That the emergence of antimicrobial resistance is a challenge that must be faced urgently has been recently recognised [1] and, as a result, there is an ongoing need for new antibacterial drugs [2,3,4]. Unfortunately, this realisation has come at a time when the antibacterial pipeline is poorly populated [5], and key pharmaceutical players are exiting the area. The tetramic acid core occurs in many natural products with antibacterial activity [6,7] and although the core tetramic motif itself tends to have no antibacterial activity [8], tetramates modified with acyl side chains can exhibit high levels of antibacterial (generally Gram-positive) activity [9,10]. Of interest, however, is whether the acyltetramate system is the key pharmacophore responsible for the observed biological activity, and whether the Gram-positive potency can be extended to Gram-negative activity. A probable requirement for success in the latter regard would be the introduction of significantly higher levels of polarity and hydrophilicity to improve membrane permeation, a parameter of crucial importance for antibacterials [11,12], which are generally more polar and different to many other biologically active systems [13]. This requirement does not necessarily intersect with the requirements of Lipinski′s “rule of five” (good absorption predicted if H-bond donors < 5; H-bond acceptors < 10; molecular weight < 500; clogP < 5) which is preferred for good oral absorption in mammalian systems [14]. Some fused-ring quinolone-containing compounds which are structurally similar to tetramates, such as pyrrolo[3,4-c]quinoline-1,3-dione derivatives, exhibit inhibition of Gram-positive and Gram-negative bacteria [15] and of interest to us has been the synthesis of related fused-ring derivatives. We have reported one example of such a system [16], which was found to have a low level of Gram-negative activity, and report here an extension of that work, providing access to a small library of polycyclic heterocyclic derivatives, which also exhibit weak Gram-negative antibacterial activity.

## 2. Results and Discussion

**Scheme 1:**
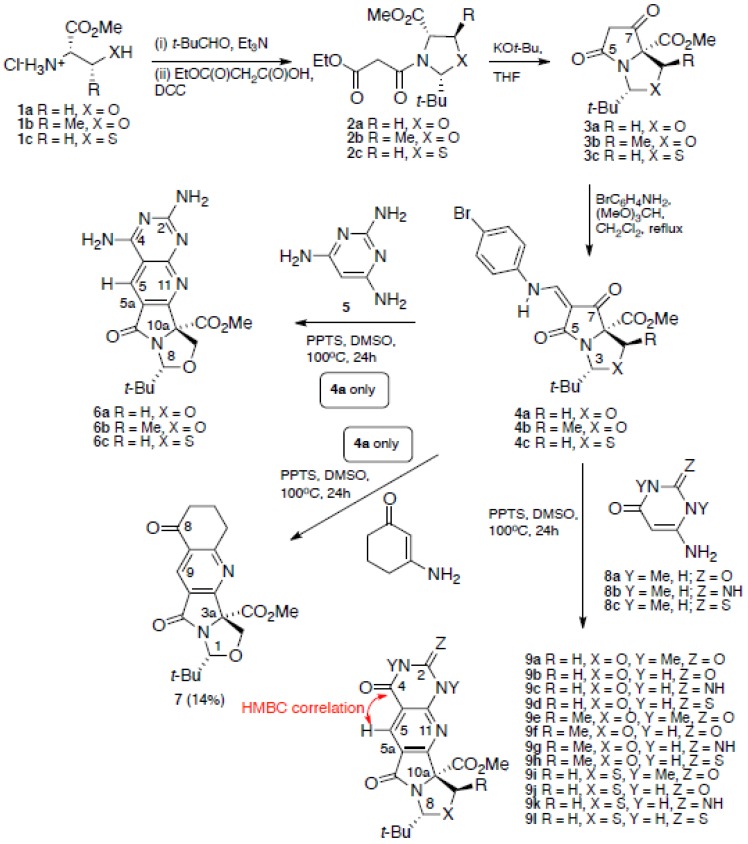
Synthetic route for key compounds.

**Figure 1:**
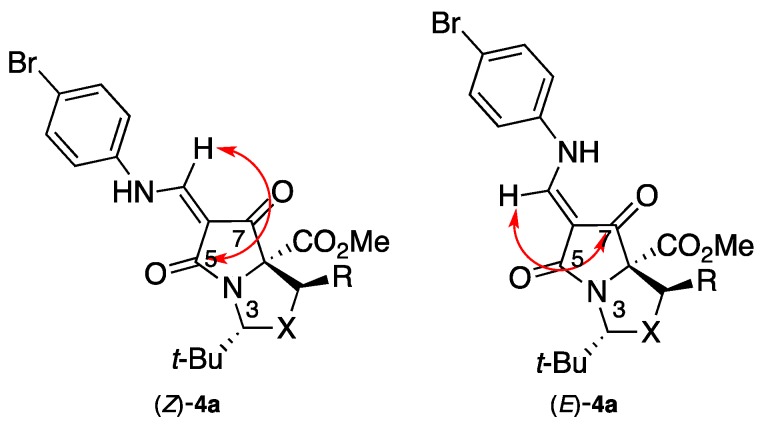
NMR characterization of key compounds.

Our approach began with tetramates **3a–c**, which we have reported previously, to be available in enantiopure form from the esters of serine **1a**, threonine **1b**, and cysteine **1c** (Scheme 1) [17,18]; this outcome arises as a result of the selective formation of *cis*-oxa(thia)zolidines **2a–c**, which chemoselectively cyclise to the product tetramates under basic conditions. Reaction of each of these with trimethyl orthoformate and *para*-bromoaniline (using a protocol which has been recently reported [19]) gave the corresponding enamines **4a–c**; these were obtained as E/Z mixtures, and the identity of the major one as Z was established by dihedral-angle-dependent coupling constants [20] from HMBC NMR experiments; thus, the intensity of the coupling between the exocyclic vinylic proton and C-5 is greater than that with C-7 in major product **(*Z*)-4a**, and vice versa in the minor product **(*E*)-4a**, clearly indicating the double bond geometry in each case (Figure 1).

Enamine **4a**, on heating with 2,4,6-triaminopyrimidine **5**, had been previously converted to tetracycle **6a** [16]; this reaction was found to give a better yield (68% compared to 47%) when conducted with the addition of pyridinium p-toluenesulfonate (PPTS), giving material which was identical to the previously reported material (NMR data recorded in CD_3_CN and MeOD respectively). Of interest was whether this approach might be extended, and the other tetramates **4b,c** were examined; upon heating them with 2,4,6-triaminopyrimidine **5**, products **6b,c** were obtained in low yield (Table 1), but their purification was problematic due to their high basicity and polarity. Additional confirmation of the identity of **6b** came from its reaction with acetic anhydride [21,22], which indicated the formation of mono and diacetylated products by HRMS analysis. We assumed that orientation of annulation was the same as we had previously reported [16] from the similar values for δH-5 in the compound series; this was confirmed from a later X-ray structure which clearly defined the product structure (vide infra).

**Figure 2:**
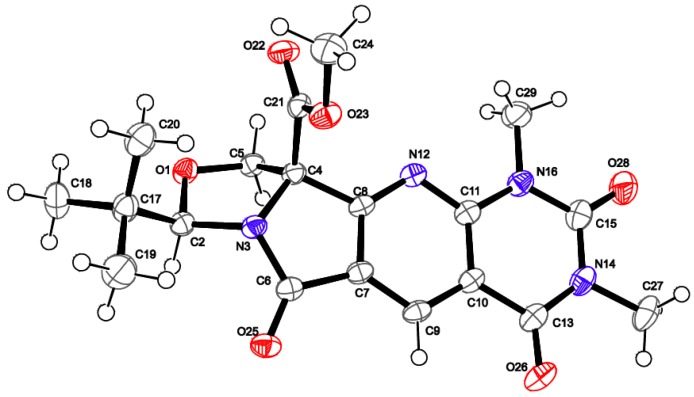
The crystal structure of compound **9a** with the ADPs shown at 50% probability. The second molecule has been omitted for clarity.

When this reaction was applied to 3-aminocyclohex-2-enone with tetramate **4a**, the corresponding product **7** was obtained in only 5% yield, which could be improved to a maximum of only 14% yield by using DMSO at 100 °C for 24 h under acid catalysis with PPTS, but of interest is that other acyclic enamine systems (3-aminocyclopent-2-en-1-one, (*Z*)-3-amino-1,3-diphenylprop-2-en-1-one, 2-aminocyclohexa-2,5-diene-1,4-dione) gave no product. Extension of this approach to other enamine-containing systems, aminopyrimidines **8a-c**, which have previously found application in annulation processes with enamines [23], gave the corresponding annulated products in modest to good yield in some cases (Table 1). The serine-derived system **4a** gave best yields (**9a–d**), and interestingly the directly related threonine-derived system reacted poorly or not at all (**9e-h**), and this presumably reflects the hindrance to reaction imposed by the C-10 methyl group over the *endo*-face of the tetramate ring. The cysteine-derived systems (**9i-j**) behaved better, and aminopyrimidine with Z = O gave a higher yield than with Z = NH, while **9l** (Z = S) gave a poor yield and could not be obtained in pure form. In the NMR spectra, similar δH-10 values in the compound series were strongly suggestive of similar structure; HMBC experiments also showed strong coupling between H-5 and C-4, supporting the formation of the proposed structure rather than alternative regioisomeric products. In the case of *N*-Me derivative **9a**, the structural assignment was further confirmed from single crystal X-ray analysis (Figure 2).

The use of enaminones for ring annulation by nucleophilic displacement has been widely reported [24,25] but, of interest, firstly, is the lack of reactivity of the oxazolidine system under these conditions, probably as a result of the unfavourable ring-imine tautomeric equilibrium [26] and, secondly, the regioselectivity of the annulation process, which involves displacement of the *p*-bromoaniline unit by the β-position of the enaminone and condensation of the remaining amine group with the C-7 carbonyl; this clearly must be the thermodynamic favourable outcome of the reaction process. Moreover, better efficiency of this process for the serine-derived tetramate **4a** over the threonine- and cysteine-derived tetramates **4b**,**c** would appear to be due the greater steric hindrance in the two latter systems.

Antimicrobial assays were carried out using the hole-plate method, using both Gram-negative *Escherichia coli* and Gram-positive *Staphylococcus aureus*, at a concentration of 4mg/mL (Table 2); a combined solvent system (DMSO and water or MeOH) was required for appropriate solubility depending on the hydrophobicity of each compound. The diameter of the inhibition zone was measured twice, perpendicularly, and the bioactivity of each compound was compared to cephalosporin C; importantly, the solvent blank assays showed no bioactivity, confirming the activity of the test compounds. The selectivity of a number of the test compounds (**6a–c**, **9b**, **9e**, **9i**, **9j**) for Gram-negative (*E. coli*) activity over Gram-positive (*S. aureus*) activity is unusual and noteworthy, which we have only seen earlier with a small subset of epoxypyroglutamate systems [27,28]; by comparison, tetramate systems generally exhibit only weak Gram-negative activity but significantly stronger Gram-positive activity. Moreover, the polarity patterns are also different; in the work reported here, the most active Gram-negative systems have clogP and %PSA values in the range 1.64–1.85 and 17.6%–26.6%, respectively (Table 2), as compared to typical values of 4% and 12% observed for Gram-positive active tetramate systems [10,27]. This represents a material difference in polarity and is consistent with a requirement for higher polarity in order to achieve Gram-negative activity, to permit small molecule permeability across the Gram-negative bacterial cell wall. These hole plate bioassay results are, however, weak, and subsequent broth assays indicated MIC values of >32 μg/mL (Antimicrobial screening (broth assay) was performed by CO-ADD (The Community for Antimicrobial Drug Discovery, Brisbane, Australia), funded by the Wellcome Trust (UK) and The University of Queensland (Australia), so although these systems will not find direct therapeutic application and would require substantial optimisation for that purpose, they do indicate that selectivity in favour of Gram-negative systems is possible and not without promise.

## 3. Materials and Methods

The starting materials **1a–c**, **2a–c**, and **3a–c** were all prepared using reported methods [17,18,30]. The compounds **4a** and **6a** have been previously reported [16], but were prepared here using slightly different methods (see below).

### 3.1. General Procedure for Synthesis of Enamines

Tetramic acid **4a–c** (1 equiv.), *para*-bromoaniline (1.05 equiv.), and trimethyl orthoformate (1.05 equiv.) were dissolved in anhydrous dichloromethane (0.1 mmol/mL), and the mixture was heated under reflux for 5 h to 18.5 h. The yellow solution was cooled down to r.t. and concentrated in vacuo to give the product after column chromatography.

#### Methyl (3*R*,7a*R*)-6-{[(4-bromophenyl)amino]methylene}-3-(*tert*-butyl)-5,7-dioxodihydro-1*H*,3*H*-pyrrolo[1,2-*c*]oxazole-7a(5*H*)-carboxylate 4a

Bright yellow crystals; 1.8:1 diastereomers (3.87g, quant.); **δ_H_** (400 MHz, CDCl_3_) Major: 0.93 (9H, s, C(C*H*_3_)_3_), 3.51 (1H, d, *J* = 8.8 Hz, C-1*H*_A_H_B_), 3.77 (3H, s, OC*H*_3_), 4.82 (1H, d, *J* = 8.8 Hz, C-1H_A_*H_B_*), 4.86 (1H, s, C-3*H*), 7.08–7.11 (2H, m, ArH), 7.53–7.56 (2H, m, ArH), 8.19 (1H, d, *J* = 13.6 Hz, =C*H*), 11.00 (1H, d, *J* = 13.6 Hz, N*H*Ar); Minor: 0.92 (9H, s, C(C*H*_3_)_3_), 3.50 (1H, d, *J* = 8.8 Hz, C-1*H*_A_H_B_), 3.78 (3H, s, OC*H*_3_), 4.82 (1H, d, *J* = 8.8 Hz, C-1H_A_*H_B_*), 4.89 (1H, s, C-3*H*), 7.08–7.11 (2H, m, Ar*H*), 7.53–7.56 (2H, m, Ar*H*), 8.10 (1H, s, =C*H*), 10.88 (1H, br s, N*H*Ar); **δ_C_** (101 MHz, CDCl_3_) Major: 24.8 (C(*C*H_3_)_3_), 35.4 (*C*(CH_3_)_3_), 53.3 (O*C*H_3_), 68.5 (*C*-1), 78.1 (*C*-7a), 98.3 (*C*-3), 99.1 (*C*-6), 119.3, 120.2, 133.4 (all Ar*C*), 136.8 (Ar*C*), 146.4 (=*C*H), 168.3 (*C*O_2_Me), 177.7 (*C*-5), 189.1 (*C*-7); Minor: 24.86 (C(*C*H_3_)_3_), 35.43 (*C*(CH_3_)_3_), 53.33 (O*C*H_3_), 68.40 (*C*-1), 77.39 (*C*-7a), 98.35 (*C*-3), 100.28 (*C*-6), 119.41, 120.38, 133.38, 136.79 (all Ar*C*), 147.23 (=*C*H), 168.37 (*C*O_2_Me), 174.35 (*C*-5), 192.50 (*C*-7); **m/z** (ESI^+^) 437.1 ([M+H]^+^); **HRMS** (ESI^+^) found 437.0707, 439.0686, 440.0720 ([M+H]^+^) requires 437.0707, 439.0686, 440.0720; **ν_max_/cm^-1^** 2957 (C–H), 2870 (C–H), 1748 (C=O), 1708 (C=O) 1656, 1620 (C=O); **m.p.** 111–114 °C; **R*_f_*** 0.2 (2:8 EtOAc:petroleum ether).

#### Methyl (1*R*,3*R*,7a*R*)-6-{[(4-bromophenyl)amino]methylene}-3-(*tert*-butyl)-1-methyl-5,7-dioxodihydro-1*H*,3*H*-pyrrolo[1,2-*c*]oxazole-7a(5*H*)-carboxylate 4b

Light yellow crystals; 1.8:1 diastereomers (1.66 g, 88%); **δ_H_** (400 MHz, CDCl_3_) Major: 0.92 (9H, s, C(C*H*_3_)_3_), 1.07 (3H, d, *J* = 6.8 Hz, C-1C*H_3_*), 3.76 (3H, s, OC*H*_3_), 4.97 (1H, s, C-3*H*), 5.10–5.16 (1H, m, C-1*H*), 7.07–7.11 (2H, m, ArH), 7.53–7.56 (2H, m, ArH), 8.14 (1H, d, *J* = 13.4 Hz, =C*H*), 10.91 (1H, d, *J* = 13.4 Hz, N*H*Ar); Minor: 0.91 (9H, s, C(C*H*_3_)_3_), 1.04 (3H, d, *J* = 6.8 Hz, C-1C*H_3_*), 3.76 (3H, s, OC*H*_3_), 4.99 (1H, s, C-3*H*), 5.10-5.16 (1H, m, C-1*H*), 7.07–7.11 (2H, m, Ar*H*), 7.53–7.56 (2H, m, Ar*H*), 8.05 (1H, br s, =C*H*), 10.86 (1H, br s, N*H*Ar); **δ_C_** (101 MHz, CDCl_3_) Major: 14.9 (C-1*C*H_3_), 24.9 (C(*C*H_3_)_3_), 35.5 (*C*(CH_3_)_3_), 53.3 (O*C*H_3_), 74.9 (*C*-1), 81.1 (*C*-7a), 95.7 (*C*-3), 100.8 (*C*-6), 119.2, 120.0, 133.4, 136.9 (all Ar*C*), 145.5 (=*C*H), 168.6 (*C*O_2_Me), 177.3 (*C*-5), 189.4 (*C*-7); Minor: 15.0 (C-1*C*H_3_), 24.9 (C(*C*H_3_)_3_), 35.5 (*C*(CH_3_)_3_), 53.3 (O*C*H_3_), 74.7 (*C*-1), 80.3 (*C*-7a), 95.8 (*C*-3), 102.0 (*C*-6), 119.3 (Ar*C*), 120.2 (Ar*C*), 133.4 (Ar*C*), 136.9 (Ar*C*), 146.1 (=*C*H), 168.7 (*C*O_2_Me), 174.0 (*C*-5), 192.8 (*C*-7); **m/z** (ESI^−^) 449.0 ([M-H]^−^); **HRMS** (ESI^−^) found 449.0723, 451.0702, 452.0734, 450.0754 ([M+H]^−^) requires 449.0718, 451.0697, 452.0731, 450.0751; **ν_max_/cm^-1^** 2958 (C–H), 2871 (C–H), 1747 (C=O), 1708 (C=O), 1656, 1620 (C=O); **m.p.** 90–92 °C; **R*_f_*** 0.5 (1:5 EtOAc:petroleum ether).

#### Methyl (3*R*,7a*R*)-6-{[(4-bromophenyl)amino]methylene}-3-(*tert*-butyl)-5,7-dioxodihydro-1*H*,3*H*-pyrrolo[1,2-*c*]thiazole-7a(5*H*)-carboxylate 4c

Bright yellow crystals; 1.9:1 diastereomers (0.45 g, 30%); **δ_H_** (400 MHz, CDCl_3_) Major: 0.98 (9H, s, C(C*H*_3_)_3_), 2.87 (1H, d, *J* = 11.4 Hz, C-1*H*_A_H_B_), 3.77 (3H, s, OC*H*_3_), 3.80 (1H, br s, C-1H_A_*H_B_*), 5.13 (1H, s, C-3*H*), 7.07–7.10 (2H, m, Ar*H*), 7.52–7.56 (2H, m, Ar*H*), 8.14 (1H, d, *J* = 13.5 Hz, =C*H*), 10.94 (1H, d, *J* = 13.5 Hz, N*H*Ar); Minor: 0.97 (9H, s, C(C*H*_3_)_3_), 2.86 (1H, d, *J* = 11.4 Hz, C-1*H*_A_H_B_), 3.77 (3H, s, OC*H*_3_), 3.79 (1H, br s, C-1H_A_*H_B_*), 5.18 (1H, s, C-2*H*), 7.07–7.10 (2H, m, Ar*H*), 7.52–7.56 (2H, m, Ar*H*), 8.05 (1H, d, *J* = 13.5 Hz, =C*H*), 10.76 (1H, d, *J* = 13.5 Hz, N*H*Ar); **δ_C_** (101 MHz, CDCl_3_) Major: 26.6 (C(*C*H_3_)_3_), 33.7 (*C*-1), 36.9 (*C*(CH_3_)_3_), 53.4 (O*C*H_3_), 73.7 (*C*-3), 82.7 (*C*-7a), 97.7 (*C*-6), 119.2, 120.0, 133.4, 136.9 (all Ar*C*), 145.9 (=*C*H), 168.3 (*C*O_2_Me), 175.3 (*C*-5), 189.8 (*C*-7); Minor: 26.6 (C(*C*H_3_)_3_), 33.9 (*C*-4), 37.0 (*C*(CH_3_)_3_), 53.4 (O*C*H_3_), 73.8 (*C*-2), 81.8 (*C*-5), 98.9 (*C*-7), 119.3, 120.2, 133.4, 136.9 (all Ar*C*), 146.8 (*C*-10), 168.4 (*C*O_2_Me), 172.2 (*C*-5), 193.1 (*C*-7); **m/z** (ESI^+^) 453.0 ([M+H]^+^); **HRMS** (ESI^+^) found 453.0477, 455.0456, 456.0491, 454.0512 ([M+H]^+^) requires 453.0478, 455.0458, 456.0491, 454.0512; **ν_max_/cm^−1^** 3025(N–H), 2953 (C–H), 1748 (C=O), 1707 (C=O) 1622 (C=O); **m.p.** 164–167 °C; **R*_f_*** 0.3 (15:85 EtOAc:petroleum ether).

### 3.2. General Procedure for Annulation Process

Respective tetramate enamine **4a–c** (1 equiv.) was dissolved in anhydrous DMSO, along with corresponding amine and/or catalytic PPTS. The solution was stirred under nitrogen at 100 °C until all starting material was consumed by TLC analysis. After cooling to r.t. the mixture was diluted with EtOAc and washed with saturated brine. The organic layer was separated, dried over MgSO_4_, filtered, and concentrated in vacuo. The product was purified by flash column chromatography.

#### Methyl (8*R*,10a*S*)-2,4-diamino-8-(*tert*-butyl)-6-oxo-6*H*,8*H*-oxazolo[3′′,4′′:1′,5′] pyrrolo[3′,4′:5,6]pyrido[2,3-*d*]pyrimidine-10a(10*H*)-carboxylate 6a

2,4,6-Triaminopyrimidine (1.3 equiv.) and PPTS (0.2 equiv.) were used in DMSO (0.032 mmol/mL), reaction time 24 h. Pale pink solid (0.038 g, 68%); **δ_H_** (400 MHz, CD_3_CN) 0.95 (9H, s, C(C*H_3_)*_3_), 3.51 (1H, d, *J* = 8.8 Hz, C-10*H_A_*H_B_), 3.68 (3H, s, OC*H*_3_), 4.89 (1H, s, C-8*H*), 4.92 (1H, d, *J* = 8.8 Hz, C-10H_A_*H_B_*), 5.87 (2H, br s, N*H*_2_), 6.61 (2H, br s, N*H*_2_), 8.53 (1H, s, C-5*H*); **δ_C_** (101 MHz, CD_3_CN) 25.2 (-C(*C*H_3_)_3_), 36.0 (-*C*(CH_3_)_3_), 53.8 (-O*C*H_3_), 70.1 (*C*-10), 76.5 (*C*-10a), 98.3 (*C*-8), 106.5, 118.0 (*C*-5a, *C*-4a), 131.6 (*C*-5), 162.0, 163.6, 165.3, 165.5 (*C*-10b, *C*-4, *C*-2, *C*-11a), 170.1 (*C*O_2_Me), 176.6 (*C*-6); **m/z** (ESI^+^) 373.2 ([M+H]^+^); **HRMS** (ESI^+^) found 373.1619 ([M+H]^+^) requires 373.1619; **ν_max_/cm^-1^** 3338 (N–H), 3179 (N–H), 2958 (C–H), 1716 (C=O), 1610 (C=O), 1549 and 1479 (C=C aromatics); **m.p.** 215–220 °C [DEC]; **R*_f_*** 0.31 (1:10 MeOH:CHCl_3_); **[α]_D_^25^** +231.1 (c=0.7 in MeOH).

#### Methyl (8*R*,10*R*,10a*S*)-2,4-diamino-8-(*tert*-butyl)-10-methyl-6-oxo-6*H*,8*H*-oxazolo[3′′,4′′:1′,5′]pyrrolo[3′,4′:5,6]pyrido[2,3-*d*]pyrimidine-10a(10*H*)-carboxylate 6b

2,4,6-Triaminopyrimidine (7.5 equiv.) was used in DMSO (0.032 mmol/mL), reaction time 24 h. Pale yellow solid (0.0044 g, 10%); **δ_H_** (400 MHz, CD_3_CN) 0.68 (3H, d, *J* = 6.7 Hz, C-10C*H_3_*), 0.94 (9H, s, C(C*H_3_)*_3_), 3.66 (3H, s, OC*H*_3_), 4.97 (1H, s, C-8*H*), 5.29 (1H, q, *J* = 6.7 Hz, C-10*H*), 5.75 (2H, br s, N*H*_2_), 6.52 (2H, br s, N*H*_2_), 8.52 (1H, s, C-5*H*); **δ_C_** (101 MHz, CD_3_CN) 15.2 (C-10*C*H_3_), 25.2 (-C(*C*H_3_)_3_), 36.1 (-*C*(CH_3_) _3_), 53.8(-O*C*H_3_), 75.6(*C*-10), 79.4 (*C*-10a), 95.6 (*C*-8), 106.3, 131.1 (*C*-5a, *C*-4a), 131.0 (*C*-5), 165.4, 165.5, 165.6, 168.8 (*C*-10b, *C*-4, *C*-2, *C*-11a), 170.4 (*C*O_2_Me), 173.0 (*C*-6); **m/z** (ESI^+^) 373.2 ([M+H]^+^); **HRMS** (ESI^+^) found 387.1776 ([M+H]^+^) requires 387.1775; **ν_max_/cm^-1^** 3724 (N–H), 3647 (N–H), 2973 (C-H), 1717 (C=O), 1606 (C=O), 1546 and 1469 (C=C aromatics); **m.p.** 200–203 °C; **R*_f_***: 0.33 (1:10 MeOH:CHCl_3_); **[α]_D_^25^** -15.8 (c=0.3in MeOH).

#### Methyl (8*R*,10a*R*)-2,4-diamino-8-(*tert*-butyl)-6-oxo-6*H*,8*H*-thiazolo[3′′,4′′:1′,5′] pyrrolo[3′,4′:5,6]pyrido[2,3-*d*]pyrimidine-10a(10*H*)-carboxylate 6c

2,4,6-Triaminopyrimidine (2.0 equiv.) was used in DMSO (0.032 mmol/mL), reaction time 24 h. Pale yellow solid (0.015 g, 13%); **δ_H_** (400 MHz, CD_3_CN) 1.01 (9H, s, C(C*H_3_)*_3_), 2.87 (1H, d, *J* = 11.3 Hz, C-10*H_A_*H_B_), 3.66 (3H, s, OC*H*₃), 3.90 (1H, d, *J* = 11.3Hz, C-10H_A_*H_B_*), 5.14 (1H, s, C-8*H*), 5.71 (2H, br s, N*H*_2_), 6.47 (2H, br s, N*H*_2_), 8.48 (1H, s, C-5*H*); **m/z** (ESI^+^) 389.1 ([M+H]^+^); **HRMS** (ESI^+^) found 389.1387 ([M+H]^+^) requires 389.1390; **ν_max_/cm^−1^**: 3348 (N-H), 3191 (N-H), 2955 (C-H), 1740 (C=O), 1620 (C=O), 1542 and 1464 (C=C aromatics); **m.p.** 230–233 °C [dec.]; **R*_f_*** 0.27 (1:10 MeOH:CHCl_3_).

#### Methyl-(1*S*,3a*S*)-1-(*tert*-butyl)-8,10-dioxo-5,7,8,10-tetrahydro-1*H*,3*H*-oxazolo [3′,4′:1,2]pyrrolo[3,4-*b*]quinoline-3a(6*H*)-carboxylate 7

3-Amino-2-cyclohexen-1-one (1.3 equiv.) and PPTS (0.2 equiv.) were used in DMSO (0.018 mmol/mL), reaction time 24 h. Colourless oil (0.0058 g, 14%); **δ_H_** (400 MHz, CDCl_3_) 1.00 (9H, s, -C(C*H*_3_)_3_), 2.19–2.27 (2H, m, C-6*H_A_H_B_*), 2.74 (2H, dd, *J* = 7.7 Hz, 5.5 Hz, C-7*H_A_H_B_*), 3.17–3.29 (2H, m, C-5*H_A_H_B_*), 3.40 (1H, d, *J* = 8.7 Hz, C3*H*_A_H_B_), 3.75 (3H, s, CO_2_C*H*_3_), 4.95 (1H, s, C-1*H*), 5.11 (1H, d, *J* = 8.7 Hz, C3H_A_*H*_B_), 8.69 (1H, s, C-9*H*); **δ_C_** (101 MHz, CDCl_3_) 21.5 (*C*-6), 24.9 (C(*C*H_3_)_3_), 33.3 (*C*-5), 35.7 (*C*(CH_3_)_3_), 38.5 (*C*-7), 53.6 (-O*C*H_3_), 69.8 (*C*-3), 97.8 (*C*-1), 125.4 (*C*-9a), 129.7 (*C*-10), 133.0 (C-8a), 166.4 (*C*-3b), 168.6 (*C*O_2_Me), 169.1 (*C*-4a), 171.6 (*C*-10), 196.2 (*C*-9); **m/z** (ESI^+^) 359.4 ([M+H]^+^); **HRMS** (ESI^+^) found 359.3603, (MH^+^) requires 359.3602; **ν_max_/cm^−1^** 2956(C–H), 2928 (C–H), 1751 (C=O), 1720 (C=O), 1694 (C=O); **R*_f_*** 0.14 (1:2 EtOAc: petroleum ether); **[α]_D_^25^** +113.2 (c = 0.4 in chloroform).

#### Methyl (8*R*,10a*S*)-8-(*tert*-butyl)-1,3-dimethyl-2,4,6-trioxo-1,3,4,6-tetrahydro- 2*H*,8*H*-oxazolo[3′′,4′′:1′,5′]pyrrolo[3′,4′:5,6]pyrido[2,3-*d*]pyrimidine-10a(10*H*)-carboxylate 9a

6-Amino-1,3-dimethyluracil (4.5 equiv.) was used in DMSO (0.018 mmol/mL), reaction time 20 h. White solid (0.041g, 45%); **δ_H_** (400 MHz, CDCl_3_) 0.98 (9H, s, C(C*H_3_*)_3_), 3.41 (1H, d, *J* = 8.7 Hz, C-10*H_A_*H_B_), 3.47 (3H, s, NC*H*_3_), 3.70 (3H, s, NC*H*_3_), 3.72 (3H, s, OC*H₃*), 4.94 (1H, s, C-8*H*), 5.08 (1H, d, *J* = 8.7 Hz, C-10H_A_*H_B_*), 8.82 (1H, s, C-5*H*); **δ_C_** (101 MHz, CDCl_3_) 24.9 (C(*C*H_3_)_3_), 28.9 (N*C*H_3_), 30.5 (N*C*H_3_), 35.6 (*C*(CH_3_)_3_), 53.5 (O*C*H_3_), 69.6 (*C*-10), 75.8 (*C-*10a), 97.8 (*C*-8), 112.2 (*C*-4a), 121.4 (*C-*5a), 135.9 (*C*-5), 150.9 (*C*-2), 154.3, 160.5 (*C*-4, *C*-11a), 167.8 (*C*-10b), 168.2 (*C*O_2_Me), 171.1 (*C*-6); **HRMS** (ESI^+^) found 403.16201 ([M+H]^+^) requires 403.16121; **ν_max_/cm^-1^** 1754 (C=O), 1715 (C=O), 1670 (C=O), 1609 (C=O); **m.p.** 193–194 °C; **R_f_** (1:2 EtOAc:petroleum ether) 0.35; **[α]_D_^25^** +70.4 (c = 1 in chloroform). Low temperature [31] single crystal X-ray diffraction data were collected using a (Rigaku) Oxford Diffraction SuperNova diffractometer (λ=1.5418 Å). Raw frame data were reduced using CrysAlisPro and the structures were solved using “Superflip” [32] before refinement with CRYSTALS [33,34] as per the SI (CIF). Full refinement details are given in the Supporting Information (CIF). Single crystal X-ray diffraction data for compound **9a**: M_r_ = 402.41; Monoclinic, P2_1_; *a* = 12.1913(2), *b* = 13.3572(2), *c* = 12.7427(3) Å, β = 109.031(2)º, V = 1961.62(7) Å^3^; T = 150 K; Z = 4; µ = 0.864 mm^−1^. Reflections collected = 21772; independent reflections = 7313 (Rint = 0.029); R values [I > 2σ(I), 11067 reflections]: R1 = 0.0301, wR2 = 0.0718. The Flack *x* parameter [35,36] refined to −0.20 (11). Bayesian analysis of the Bijvoet pair [37] gave the Hooft *y* parameter as −0.059(71) and the probability that the structure was the correct hand was >99.9% given that the crystal is enantiopure. Crystallographic data have been deposited with the Cambridge Crystallographic Data Centre as supplementary publication No. 1517235 (www.ccdc.cam.ac.uk/conts/retrieving.html).

#### Methyl (8*R*,10a*S*)-8-(*tert*-butyl)-1,3-dimethyl-2,4,6-trioxo-1,3,4,6-tetrahydro-2*H*,8*H*-oxazolo[3′′,4′′:1′,5′]pyrrolo[3′,4′:5,6]pyrido[2,3-*d*]pyrimidine-10a(10*H*)-carboxylate 9b

6-Aminouracil (3.0 equiv.) was used in DMSO (0.032 mmol/mL), reaction time 24 h. White solid (0.0379 g, 82%); **δ_H_** (400 MHz, MeOD) 0.97 (9H, s, C(C*H*_3_)_3_), 3.57 (1H, d, *J* = 8.6 Hz, C-10*H*_A_H_B_), 3.71 (3H, s, OC*H*_3_), 4.92 (1H, s, C-8*H*), 4.96 (1H, d, *J* = 8.6 Hz, C-10H_A_*H*_B_), 8.61 (1H, s, C-5*H*); **δ_C_** (101 MHz, MeOD) 23.9 (C(*C*H_3_)_3_), 35.0 (*C*(CH_3_)_3_), 52.5 (O*C*H_3_), 56.9 (*C*-10), 68.8 (*C*-10a), 75.6 (*C*-8), 111.8, 121.1 (*C*-5a, *C*-4a), 134.1 (*C*-5), 150.5, 156.1, 161.9, 168.1 (*C*-10b, *C*-4, *C*-2, *C*-11a), 168.2 (*C*O_2_Me), 171.7 (*C*-6); **m/z** (ESI^−^) 373.1 ([M-H]^−^); **HRMS** (ESI^+^) found 375.1299, (MH^+^) requires 375.1299; **ν_max_/cm^-1^** 3552 (N–H), 3194 (N–H), 1717 (C=O), 1612 (C=O); **m.p.** 190–192 °C; **R*_f_*** 0.43 (1:10 MeOH:CHCl_3_).

#### Methyl-(8*R*,10a*S*)-2-amino-8-(*tert*-butyl)-4-hydroxy-6-oxo-6*H*,8*H*-oxazolo[3′′,4′′:1′,5′]pyrrolo[3′,4′:5,6]pyrido[2,3-*d*]pyrimidine-10a(10*H*)-carboxylate 9c

2,4-Diamino-6-hydroxypyrimidine (3.0 equiv.) was used in DMSO (0.032 mmol/mL), reaction time 24 h. White solid (0.023 g, 49%); **δ_H_** (400 MHz, DMSO) 0.91 (9H, s, C(C*H*_3_)_3_), 3.57 (1H, d, *J* = 8.6 Hz, C-10*H*_A_H_B_), 3.65 (3H, s, OC*H*_3_), 4.82 (1H, d, *J* = 8.1 Hz, C-10H_A_*H*_B_), 4.84 (1H, s, C-8*H*), 8.37 (1H, s, C-5*H*); **δ_C_** (101 MHz, DMSO): 24.7 (C(*C*H_3_)_3_), 35.0 (*C*(CH_3_)_3_), 53.2 (O*C*H_3_), 68.7 (*C*-10), 75.2 (*C*-10a), 96.5 (*C*-8), 112.9, 117.9 (*C*-5a, *C*-4a), 132.5 (*C*-10), 156.4, 156.4, 164.4, 168.0 (*C*-10b, *C*-4, *C*-2, *C*-11a), 168.8 (*C*O_2_Me), 171.8 (*C*-6); **m/z** (ESI^−^) 372.1 ([M-H]^−^); **HRMS** (ESI^−^) found 372.1313, (MH^−^) requires 372.1313; **ν_max_/cm^−1^** 3267 (N–H), 3132 (N–H), 1727 (C=O), 1705 (C=O), 1529, 1482 (C=C aromatics); **m.p.** 260–263 °C [dec.]; **R*_f_*** 0.29 (1:10 MeOH:CHCl_3_).

#### Methyl (8*R*,10a*S*)-8-(*tert*-butyl)-4,6-dioxo-2-thioxo-1,3,4,6-tetrahydro-2*H*,8*H*-oxazolo[3′′,4′′:1′,5′]pyrrolo[3′,4′:5,6]pyrido[2,3-*d*]pyrimidine-10a(10*H*)-carboxylate 9d

6-Amino-2-thiouracil (3.0 equiv.) was used in DMSO (0.032 mmol/mL), reaction time 42 h. White crystal (0.028 g, 45%); **δ_H_** (400 MHz, MeOD) 0.97 (9H, s, C(C*H*_3_)_3_), 3.60 (1H, d, *J* = 8.6 Hz, C-10*H*_A_H_B_), 3.72 (3H, s, OC*H*_3_), 4.93 (1H, s, C-8*H*), 4.97 (1H, d, *J* = 8.6 Hz, C-10H_A_*H*_B_), 8.61 (1H, s, C-5*H*); **δ_C_** (101 MHz, MeOD): 23.9 (C(*C*H_3_)_3_), 35.0 (*C*(CH_3_)_3_), 52.6 (O*C*H_3_), 68.8 (*C*-10), 75.7 (*C*-10a), 97.5 (*C*-8), 113.4 and 121.9 (*C*-5a and *C*-4a), 133.9 (*C*-5), 154.9, 159.3, 168.2, 168.3 (*C*-10b, *C*-4, *C*-2, *C*-11a), 171.4 (*C*O_2_Me), 176.6 (*C*-6); **m/z** (ESI^−^): 389.1 ([M-H]^−^); **HRMS** (ESI^−^) found 389.0925, (MH^−^) requires 389.0923; **ν_max_/cm^−1^** 1752 (C=O), 1714 (C=O), 1615 (C=C aromatics); **m.p.** 193-194 °C; **R*_f_*** 0.60 (1:10 MeOH:CHCl_3_).

#### Methyl (8*R*,10*R*,10a*S*)-8-(*tert*-butyl)-1,3,10-trimethyl-2,4,6-trioxo-1,3,4,6-tetrahydro-2*H*,8*H*-oxazolo[3′′,4′′:1′,5′]pyrrolo[3′,4′:5,6]pyrido[2,3-*d*]pyrimidine-10a(10*H*)-carboxylate 9e

6-Amino-1,3-dimethyluracil (7.0 equiv.) was used in DMSO (0.018 mmol/mL), reaction time 24 h. White solid (0.027g, 53%); **δ_H_** (400 MHz, CDCl_3_) 0.71 (3H, d, *J* = 6.7 Hz, C-10C*H_3_*) 0.96 (9H, s, C(C*H_3_*)_3_), 3.46 (3H, s, NC*H*_3_), 3.69 (3H, s, NC*H*_3_), 3.69 (3H, s, OC*H₃*), 4.99 (1H, s, C-8*H*), 5.37 (1H, q, *J* = 6.7 Hz, C-10*H*), 8.81 (1H, s, C-10*H*); **δ_C_** (101 MHz, CDCl_3_) 13.9 (C-10*C*H_3_), 23.8 (C(*C*H_3_)_3_), 27.7 (N*C*H_3_), 28.7 (N*C*H_3_), 34.5 (*C*(CH_3_)_3_), 52.3 (O*C*H_3_), 73.5 (*C*-10), 77.7 (*C-*10a), 94.0 (*C*-8), 110.9 (*C*-4a), 121.6 (*C*-5a), 134.3 (*C*-5), 149.8 (*C*-2), 153.2 and 159.2 (*C*-4, *C*-11a), 166.4 (*C*-10b), 167.5 (*C*O_2_Me), 168.9 (*C*-6); **m/z** (ESI^+^) 417.2 ([M+H]^+^); **HRMS** (ESI^+^) found 417.1768 ([M+H]^+^) requires 417.1769; **ν_max_/cm^−1^** 2960 (C–H), 1752 (C=O), 1728 (C=O), 1672 (C=O), 1609 (C=O); **m.p.** 196–199 °C; **R*_f_*** 0.31 (1:2 EtOAc: petroleum ether).

#### Methyl (8*R*,10a*R*)-8-(*tert*-butyl)-1,3-dimethyl-2,4,6-trioxo-1,3,4,6-tetrahydro-2*H*,8*H*-thiazolo[3′′,4′′:1′,5′]pyrrolo[3′,4′:5,6]pyrido[2,3-*d*]pyrimidine-10a(10*H*)-carboxylate 9i

6-Amino-1,3-dimethyluracil (11.0 equiv.) was used in DMSO (0.018 mmol/mL), reaction time 24 h. White solid (0.041 g, 36%); **δ_H_** (400 MHz, CDCl_3_) 1.04 (9H, s, C(C*H_3_*)_3_), 2.75 (1H, d, *J* = 11.3 Hz, C-10*H_A_*H_B_), 3.49 (3H, s, NC*H*_3_), 3.71 (3H, s, NC*H*_3_), 3.71 (3H, s, OC*H₃*), 3.99 (1H, d, *J* = 11.3 Hz, C-10H_A_*H_B_*), 5.19 (1H, s, C-8*H*), 8.80 (1H, s, C-5*H*); **δ_C_** (101 MHz, CDCl_3_) 26.6 (C(*C*H_3_)_3_), 28.8 (N*C*H_3_), 30.4 (N*C*H_3_), 34.1 (C-10), 53.5 (O*C*H_3_), 68.5 (*C*(CH_3_)_3_), 72.6 (*C-*8), 80.3 (*C*-10a), 112.2 (*C*-4a), 119.8 (*C*-5a), 135.7 (*C*-5), 151.0 (*C*-2), 160.4, 168.1 (*C*-4, *C*-11a), 168.1 (*C*-10b), 168.7 (*C*O_2_Me), 168.9 (*C*-6); **m/z** (ESI^+^) 419.1 ([M+H]^+^); **HRMS** (ESI^+^) found 419.1383 ([M+H]^+^) requires 419.1384; **ν_max_/cm^−1^** 2957 (C-H), 1723 (C=O), 1670 (C=O), 1612 (C=O); **m.p.** 189-192 °C; **R*_f_***: 0.37 (1:2 EtOAc:petroleum ether); **[α]_D_^25^** +65.3 (c=0.3 in chloroform).

#### Methyl (8*R*,10a*R*)-8-(*tert*-butyl)-2,4,6-trioxo-1,3,4,6-tetrahydro-2*H*,8*H*-thiazolo[3′′,4′′:1′,5′]pyrrolo[3′,4′:5,6] pyrido[2,3-*d*]pyrimidine-10a(10*H*)-carboxylate 9j

6-Aminouracil (3.0 equiv.) was used in DMSO (0.032 mmol/mL), reaction time 32 h. **δ_H_** (400 MHz, CD_3_OD) 1.02 (9H, s, C(C*H_3_*)_3_), 2.94 (1H, d, *J* = 11.3 Hz, C-10*H_A_H_B_*), 3.70 (3H, s, -CO_2_C*H*_3_), 3.88 (1H, d, *J* = 11.3 Hz, C-10H_A_*H*_B_), 5.15 (1H, s, C-8*H*), 8.57 (1H, s, C-5*H*); **δ_C_** (100 MHz, CD_3_OD) 27.2 (C(*C*H_3_)_3_), 34.2 (C-10), 38.0 (*C*(CH_3_)_3_), 53.9 (CO_2_*C*H_3_), 73.6 (C-8), 81.7 (C-10a), 113.1 (C-4a), 121.0 (C-5a), 135.2 (C-5), 151.9 (C-2), 157.4 (C-11a), 163.3 (C-4), 169.5 (*C*O_2_CH_3_), 170.5 (C-10b), 171.0 (C-6); **m/z** (ESI^-^): 389.1 ([M-H]^-^); **HRMS** (ESI^+^) observed 391.1068, C_17_H_19_N_4_O_5_S requires 391.1071; **R*_f_*** (5% MeOH in CHCl_3_) 0.41.

#### Methyl (8*R*,10a*R*)-8-(*tert*-butyl)-4,6-dioxo-2-thioxo-1,3,4,6-tetrahydro-2*H*,8*H*-thiazolo[3′′,4′′:1′,5′]pyrrolo[3′,4′:5,6]pyrido[2,3-*d*]pyrimidine-10a(10*H*)-carboxylate 9l

6-Aminouracil (3.0 equiv.) was used in DMSO (0.032 mmol/mL), reaction time 72 h. **δ_H_** (400 MHz, CD_3_OD) 1.02 (9H, s, C(C*H_3_*)_3_), 2.97 (1H, d, *J* = 11.1 Hz, C10- *H_A_*H_B_), 3.60 (3H, s, CO_2_C*H*_3_), 3.8 (1H, d, *J* = 11.1 Hz, C10- H_A_*H*_B_), 5.15 (1H, s, C-8*H*), 8.58 (1H, m, C-5*H*).

## 4. Conclusions

We have shown that ring annulation on a tetramate template provides ready access to fused ring heterocycles which exhibit weak Gram-negative activity; the structural novelty of these systems is noteworthy, and their high level of polarity gives promise for further optimization of antibacterial activity.

## Figures and Tables

**Table 1:** Yields and NMR data for key compounds.

Compound	Yield (%)	Ratio	Compound	Yield (%)	δH-5 (ppm)	Compound	Yield (%)	δH-5 (ppm)
**4a**	100	1.8:1	**6a**	68	8.53	**9a**	45	8.82
						**9b**	82	8.61
						**9c**	49	8.37
						**9d**	45	8.61
**4b**	88	1.8:1	**6b**	10	8.52	**9e**	53	8.81
						**9f**	-	-
						**9g**	-	-
						**9h**	-	-
**4c**	30	1.9:1	**6c**	13	8.48	**9i**	36	8.80
						**9j**	56	8.57
						**9k**	-	-
						**9l**	<10	8.58

**Table 2:** Antibacterial assay results for selected compounds ^a^.

Compound	Solvent system	Polar Surface Area (PSA) ^b^	Molecular Surface Area (MSA) ^b^	clogP ^b^	PSA/MSA (%) ^b^	Zone size (mm)	
						*E. coli*	*S. aureus*
6a	DMSO: H_2_O = 1:1	146.55	511.28	1.22	28.7	17	Not active
6b	DMSO: H_2_O = 1:1	146.55	540.70	1.64	27.1	17	Not active
6c	DMSO: H_2_O = 1:1	137.32	516.98	1.68	26.6	18.5	Not active
9a	DMSO: MeOH = 1:1	109.35	564.05	1.29	19.4	14.5	17
9b	DMSO: H_2_O = 1:1	126.93	499.08	1.85	25.4	17.5	Not active
9c	DMSO	140.76	506.42	1.75	27.8	16	15.5
9d	DMSO: H_2_O = 7:3	109.86	504.20	2.74	21.8	15.5	15.5
9e	DMSO: MeOH = 1:1	109.35	593.44	1.70	18.4	16.5	Not active
9i	DMSO: H_2_O = 1:1	100.12	569.60	1.74	17.6	13	Not active
9j	DMSO: MeOH = 1:1	117.2	504.20	1.44	23.2	15	12.5

^a^ Bioassay of products [29]: Microbiological assays were performed by the hole-plate method with the test organism *Staphylococcus aureus* DS267 or *E. coli* X580. Solutions (100 µL) of the compounds to be tested (4, 2, 1, 0.5 mg/mL) were loaded into wells in bioassay plates, and incubated overnight at 37 °C. The diameters of the resultant inhibition zones were measured (±0.5 mm), and compared to the positive control, Cephalosporin C. Cephalosporin C calibration and solvent blank data is provided in the Supplementary Materials Electronic Supporting Information; ^b^ Calculated using Marvin 5.3.8 (http://www.chemaxon.com).

## Supplementary Materials

The following are available online at www.mdpi.com/2079-6382/6/1/2/s1, Spectra: Electronic Supporting Information, Figure S1: *E. coli* calibration, Figure S2: *S. aureus* calibration, Table S1: Bioassary data for Ceph. C, Table S2: Bioassary data for solvents.
