# Supplementary material for: Fused-Ring Oxazolopyrrolopyridopyrimidine Systems with Gram-Negative Activity
Source: Antibiotics (Basel). 2017 Jan 13;6(1):2. doi: 10.3390/antibiotics6010002 (PMC5372982; doi:10.3390/antibiotics6010002)
Supplement: Supplementary file 1 [file antibiotics-06-00002-s001.zip › Supplementary Materials/Spectra Electronic Supporting Information.pdf]

Supporting Information

Fused Ring Oxazolopyrrolopyridopyrimidine Systems with Selective Gram Negative Activity

Yiyuan Chen, Jonathan G. Moloney, Mark G. Moloney* and Amber Thompson

Department of Chemistry, Chemistry Research Laboratory, The University of Oxford, 12 Mansfield Road, Oxford. OX1 3TA.

mark.moloney@chem.ox.ac.uk

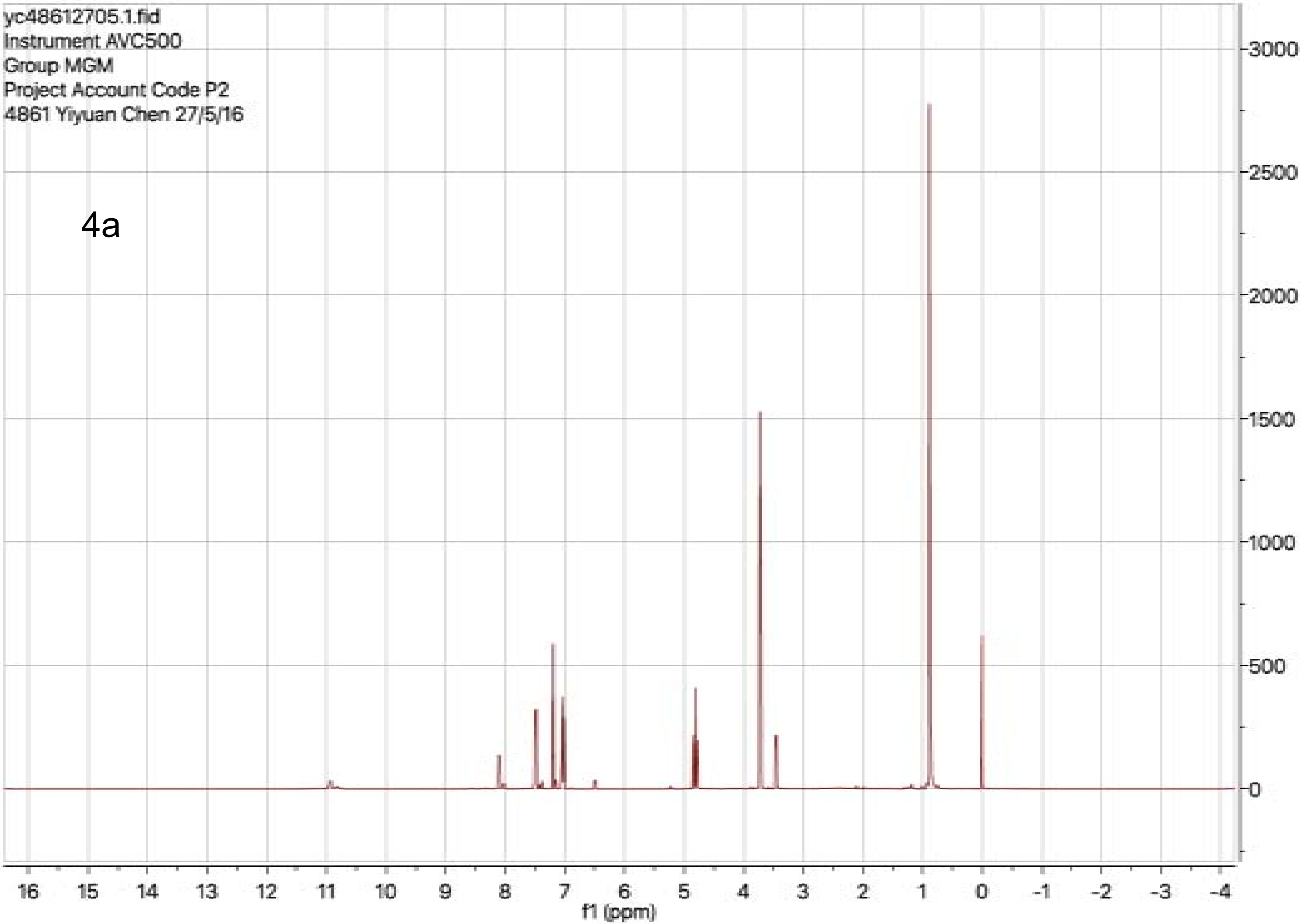
yc48612705.1.fid
Instrument AVC500
Group MGM
Project Account Code P2
4861 Yiyuan Chen 27/5/16
4a
3000
2500
2000
1500
1000
500
0
16 15 14 13 12 11 10 9 8 7 6 5 4 3 2 1 0 -1 -2 -3 -4
f1 (ppm)

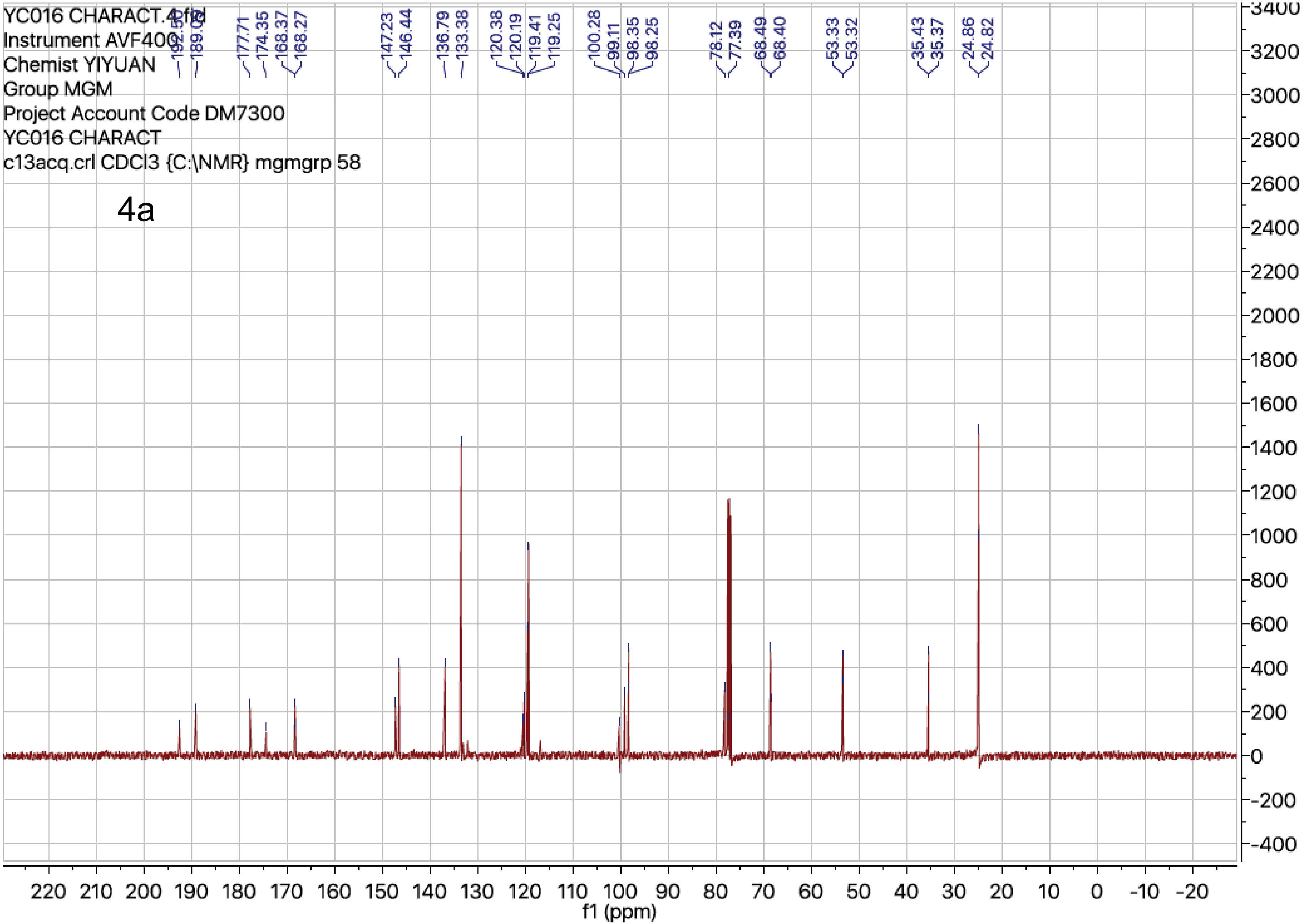
YC016 CHARACT.4.fid
Instrument AVF400
Chemist YIYUAN
Group MGM
Project Account Code DM7300
YC016 CHARACT
c13acq.crl CDCl3 {C:\NMR} mgmgrp 58
4a
177.71
174.35
168.37
168.27
147.23
146.44
136.79
133.38
120.38
120.19
119.41
119.25
100.28
99.11
98.35
98.25
78.12
77.39
68.49
68.40
53.33
53.32
35.43
35.37
24.86
24.82
f1 (ppm)

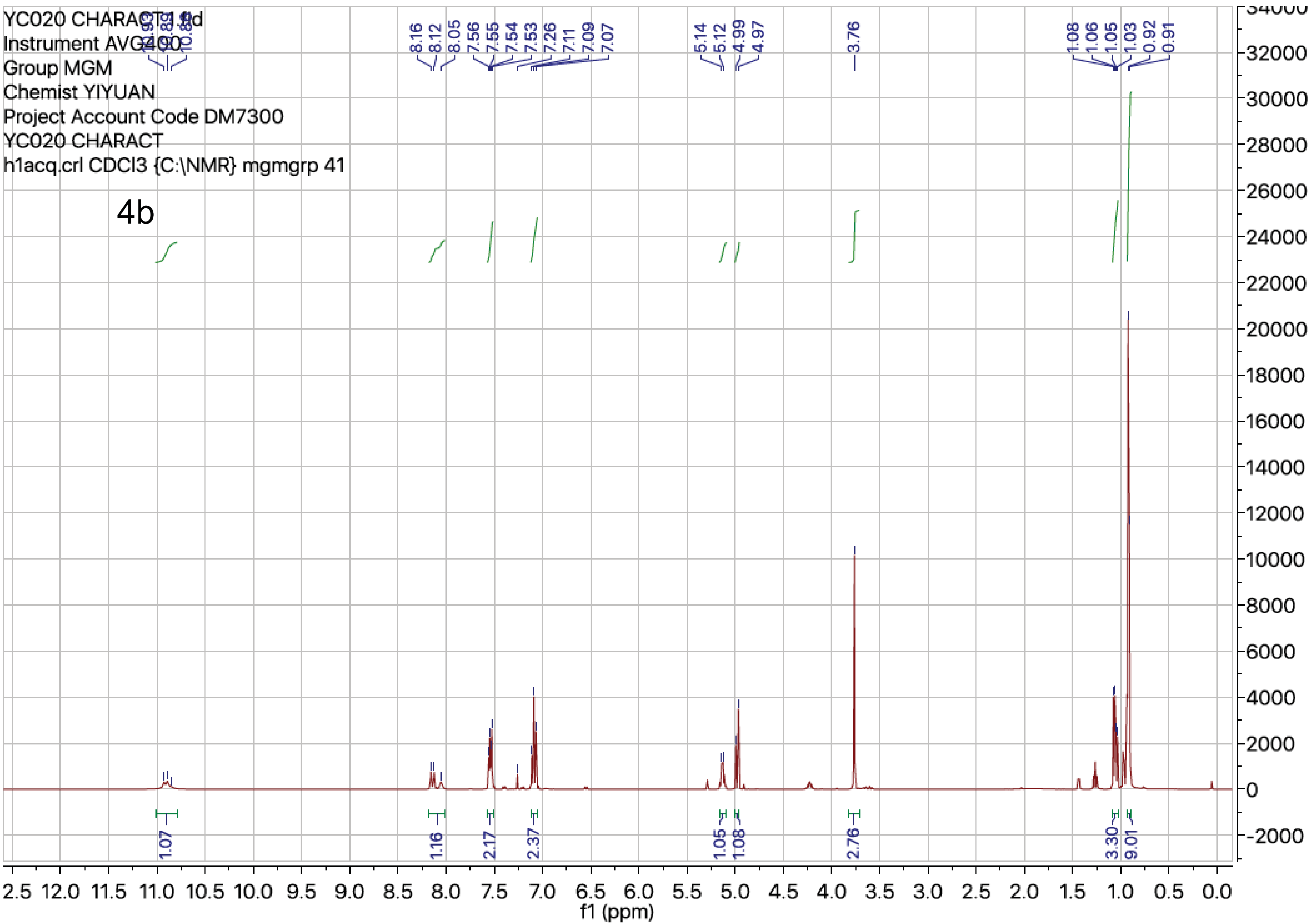
YC020 CHARACT-1.fid
Instrument AVG400
Group MGM
Chemist YIYUAN
Project Account Code DM7300
YC020 CHARACT
h1acq.crl CDCl3 {C:\NMR} mgmgrp 41
4b
8.16
8.12
8.05
7.56
7.55
7.54
7.53
7.26
7.11
7.09
7.07
5.14
5.12
4.99
4.97
3.76
1.08
1.06
1.05
1.03
0.92
0.91
1.07
1.16
2.17
2.37
1.05
1.08
2.76
3.30
9.01
f1 (ppm)

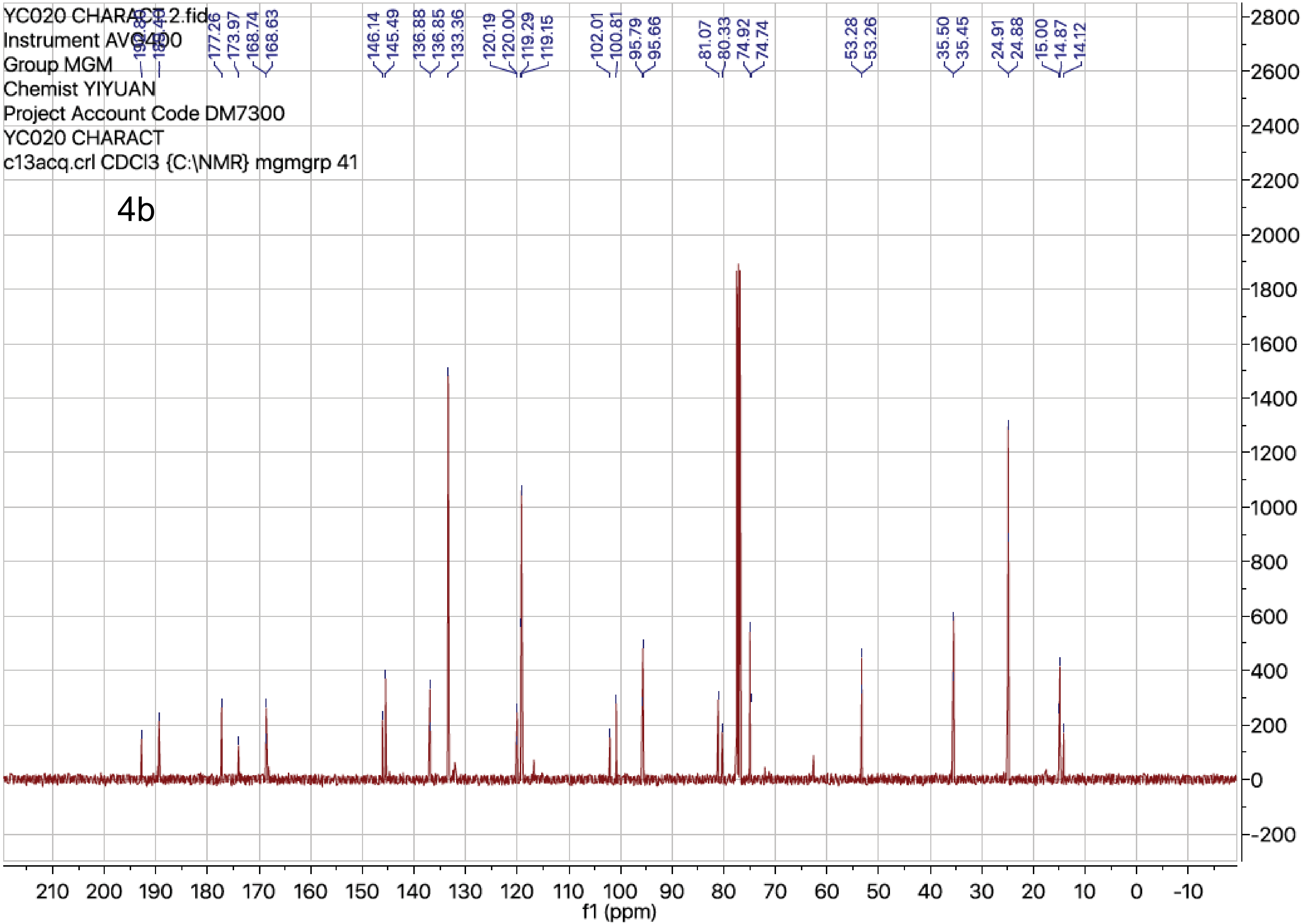
YC020 CHARACT 2.fid
Instrument AVG400
Group MGM
Chemist YIYUAN
Project Account Code DM7300
YC020 CHARACT
c13acq.crl CDCl3 {C:\NMR} mgmgrp 41
4b
177.26
173.97
168.74
168.63
146.14
145.49
136.88
136.85
133.36
120.19
120.00
119.29
119.15
102.01
100.81
95.79
95.66
81.07
80.33
74.92
74.74
53.28
53.26
35.50
35.45
24.91
24.88
15.00
14.87
14.12
f1 (ppm)

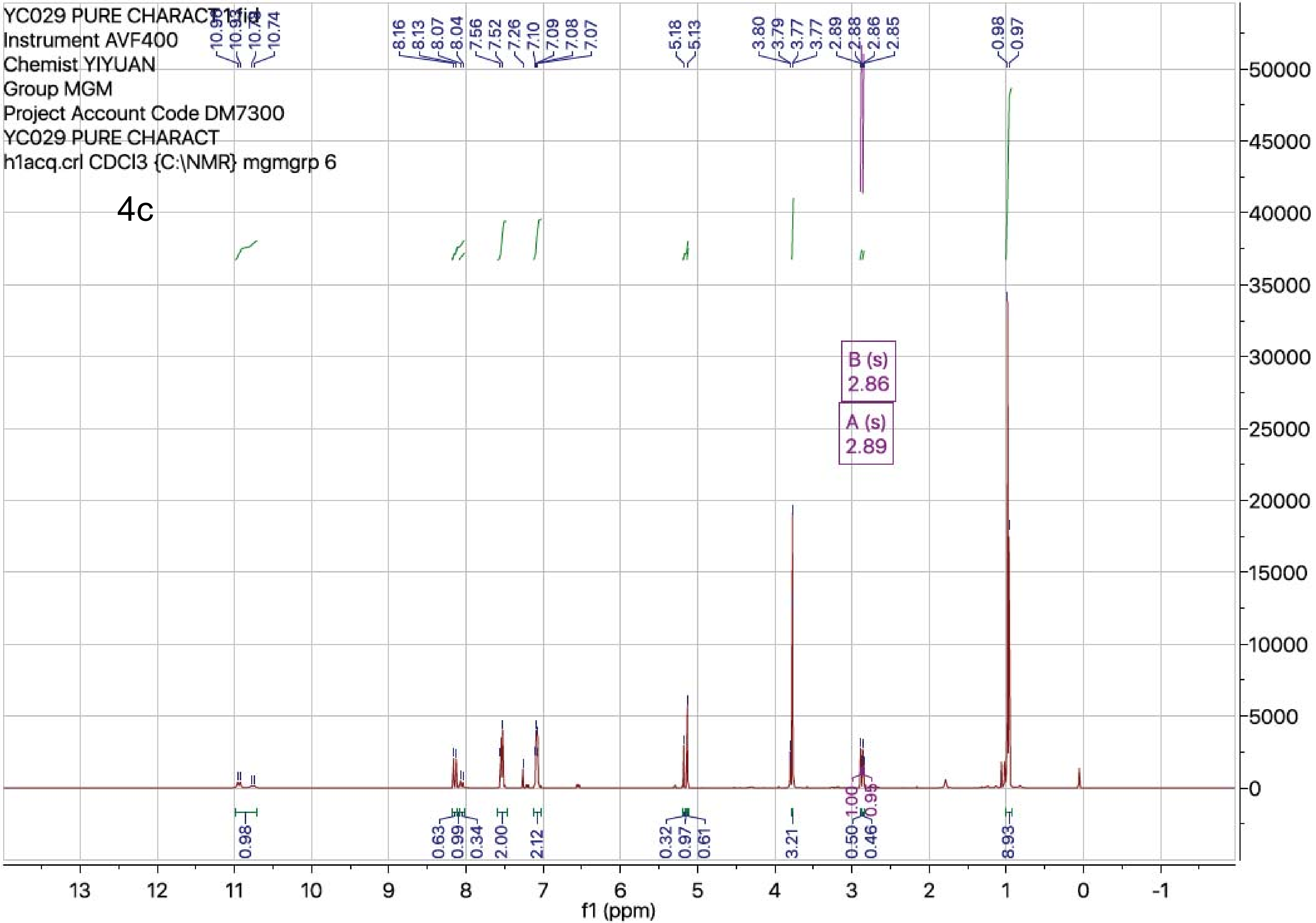
YC029 PURE CHARACT1.fid
Instrument AVF400
Chemist YIYUAN
Group MGM
Project Account Code DM7300
YC029 PURE CHARACT
h1acq.crl CDCl3 {C:\NMR} mgmgrp 6
4c
B (s) 2.86
A (s) 2.89
f1 (ppm)

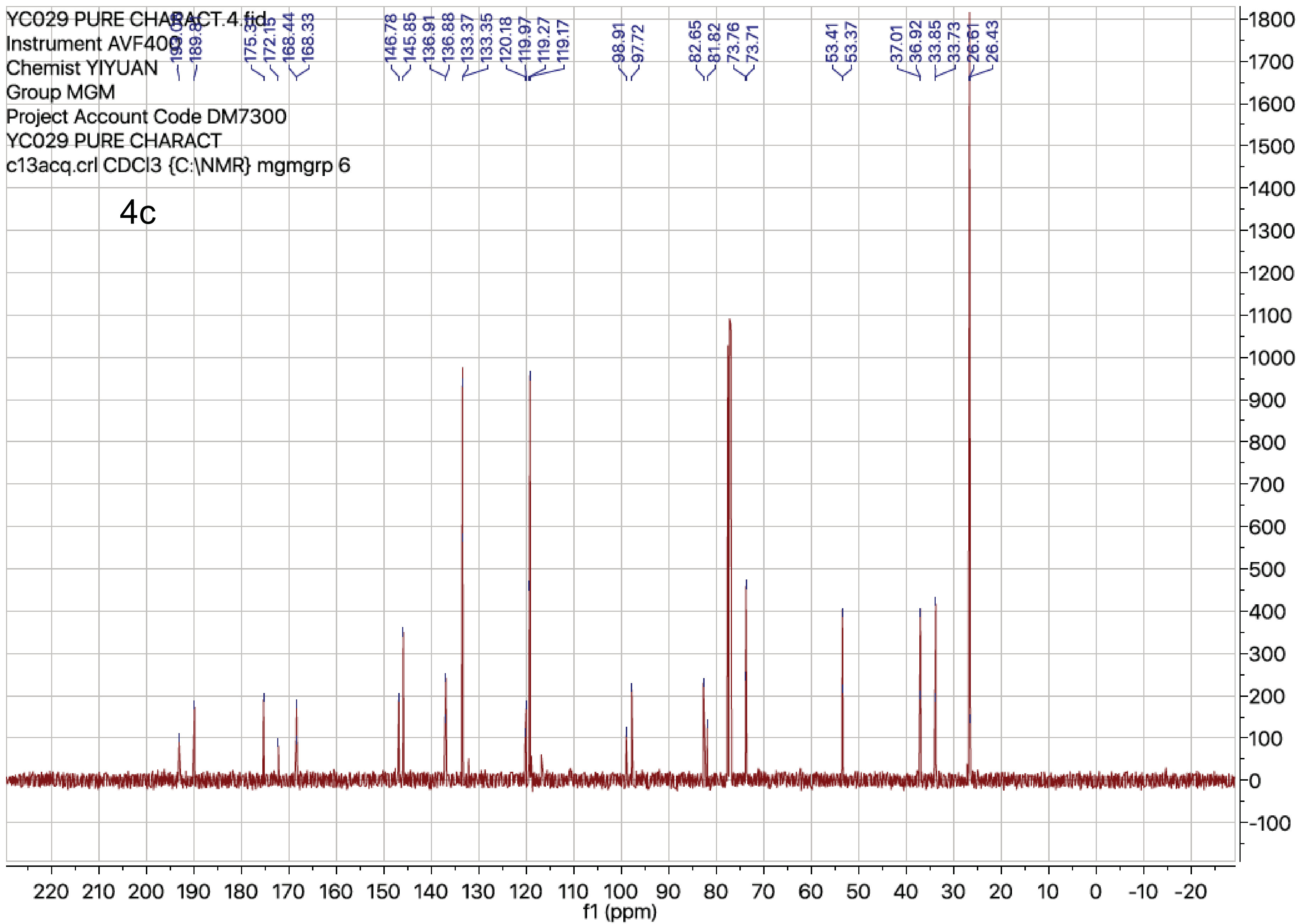
YC029 PURE CHARACT.4.fid
Instrument AVF400
Chemist YIYUAN
Group MGM
Project Account Code DM7300
YC029 PURE CHARACT
c13acq.crl CDCl3 {C:\NMR} mgmgrp 6
4c
189.8
175.3
172.15
168.44
168.33
146.78
145.85
136.91
136.88
133.37
133.35
120.18
119.97
119.27
119.17
98.91
97.72
82.65
81.82
73.76
73.71
53.41
53.37
37.01
36.92
33.85
33.73
26.61
26.43
f1 (ppm)

Instrument AVF400
Chemist YIYUAN CHEN
Group MGM
Project Account Code DM7300
YC046 CHARACT CD3CN
h1acq.crl CD3CN {C:\NMR} mgmgrp 46

6a

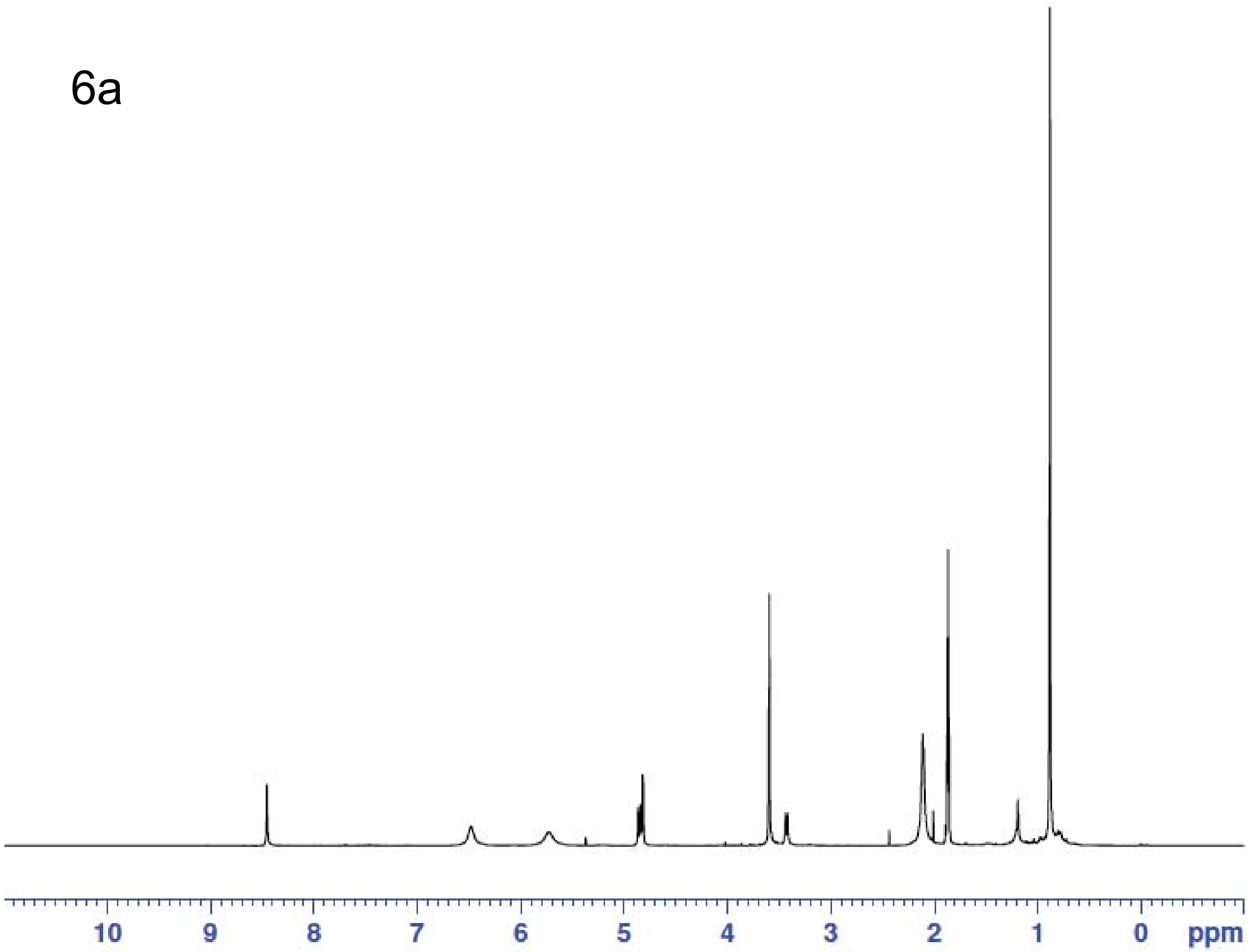

NMR@CHEM.OX

Current Data Parameters
NAME YC046 CHARAC
EXPNO 1
PROCNO 1

F2 - Acquisition Parameters
Date_ 20160505
Time 3.50 h
INSTRUM avf400
PROBHD Z108618_0533 (
PULPROG zg60
TD 65536
SOLVENT CD3CN
NS 16
DS 2
SWH 8012.820 Hz
FIDRES 0.244532 Hz
AQ 4.0894465 sec
RG 93
DW 62.400 usec
DE 6.50 usec
TE 294.9 K
D1 1.00000000 sec
TD0 1
SFO1 400.2524015 MHz
NUC1 1H
P1 19.50 usec
PLW1 16.70000076 W

F2 - Processing parameters
SI 32768
SF 400.2500418 MHz
WDW EM
SSB 0
LB 0.30 Hz
GB 0
PC 1.00

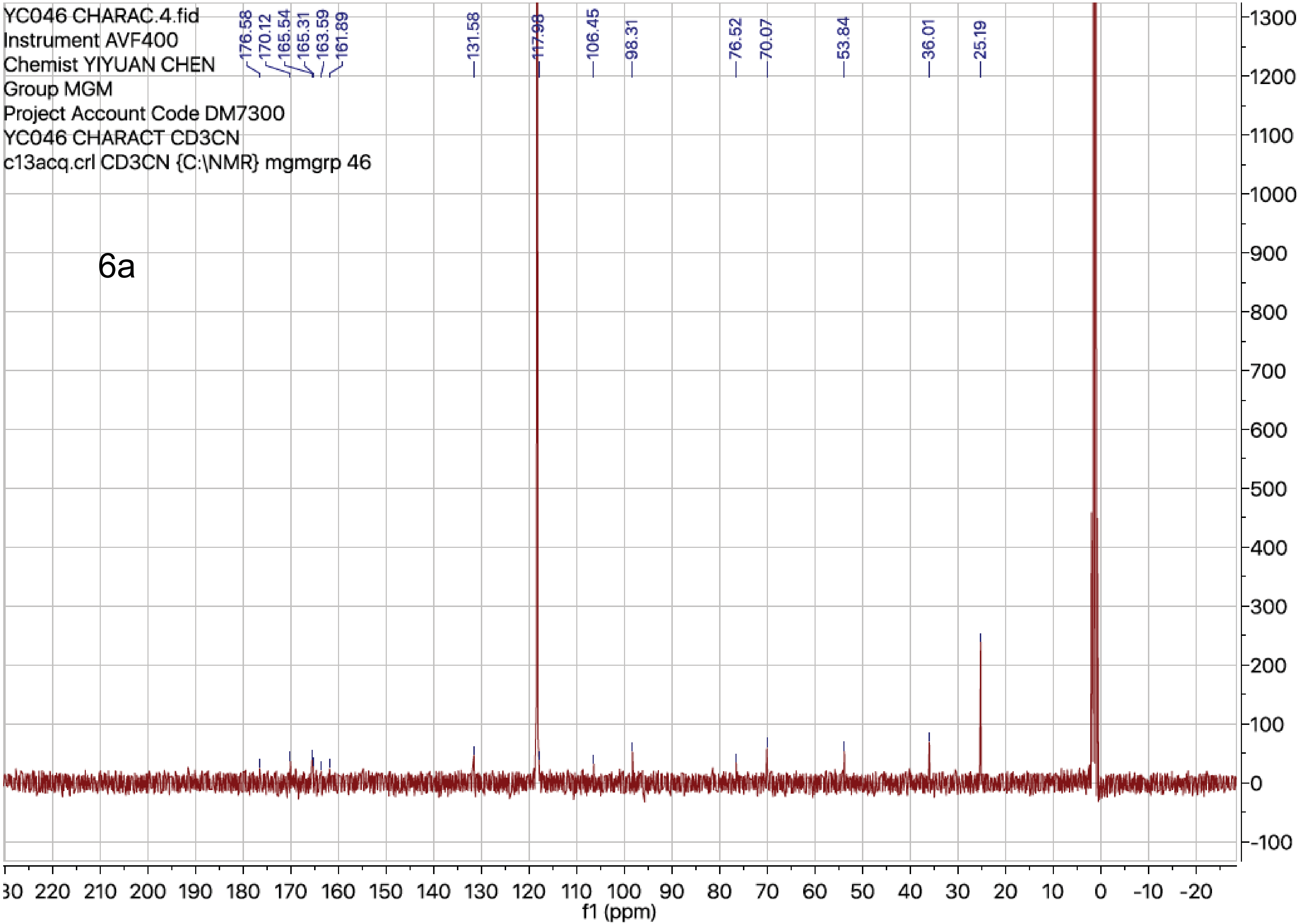
YC046 CHARAC.4.fid
Instrument AVF400
Chemist YIYUAN CHEN
Group MGM
Project Account Code DM7300
YC046 CHARACT CD3CN
c13acq.crl CD3CN {C:\NMR} mgmgrp 46
6a
176.58
170.12
165.54
165.31
163.59
161.89
131.58
117.98
106.45
98.31
76.52
70.07
53.84
36.01
25.19
f1 (ppm)

Instrument AVC500
Group MGM
Project Account Code DM7300
4568 Yiyuan Chen 5/5/16

6b

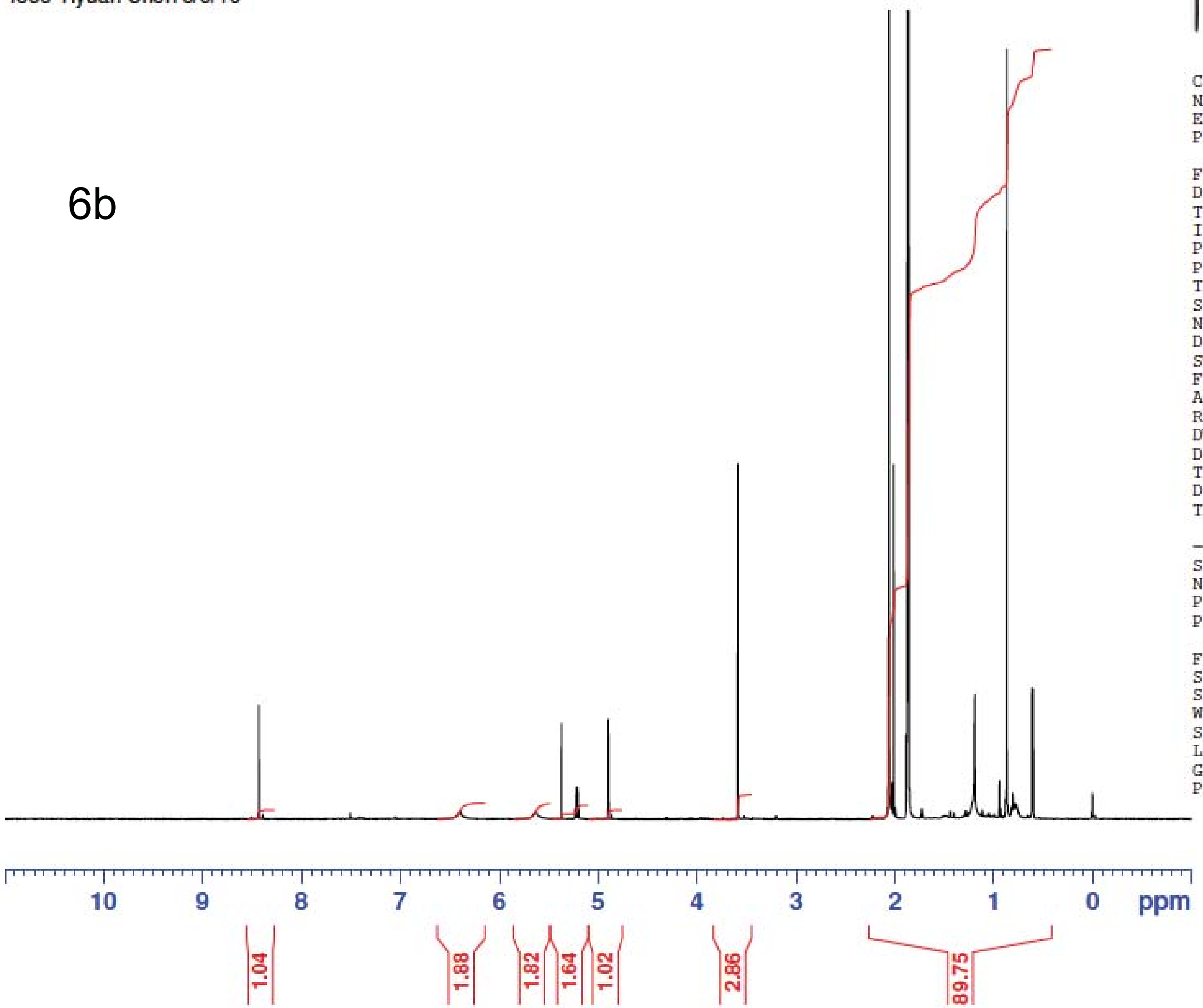

NMR@CHEM.OX

```
Current Data Parameters
NAME             yc45680505
EXPNO                     1
PROCNO                    1

F2 - Acquisition Parameters
Date_              20160506
Time                  14.05
INSTRUM              avc500
PROBHD   5 mm CPDUL 13C
PULPROG                zg30
TD                    65536
SOLVENT                CD3CN
NS                       16
DS                        4
SWH              10330.578 Hz
FIDRES            0.157632 Hz
AQ               3.1719425 sec
RG                     2.25
DW                   48.400 usec
DE                    10.00 usec
TE                    298.0 K
D1               1.00000000 sec
TD0                       1

======== CHANNEL f1 ========
SFO1          500.3030896 MHz
NUC1                     1H
P1                    15.00 usec
PLW1             7.99830008 W

F2 - Processing parameters
SI                    65536
SF            500.3000545 MHz
WDW                      EM
SSB      0
LB                     0.30 Hz
GB       0
PC                     1.00
```

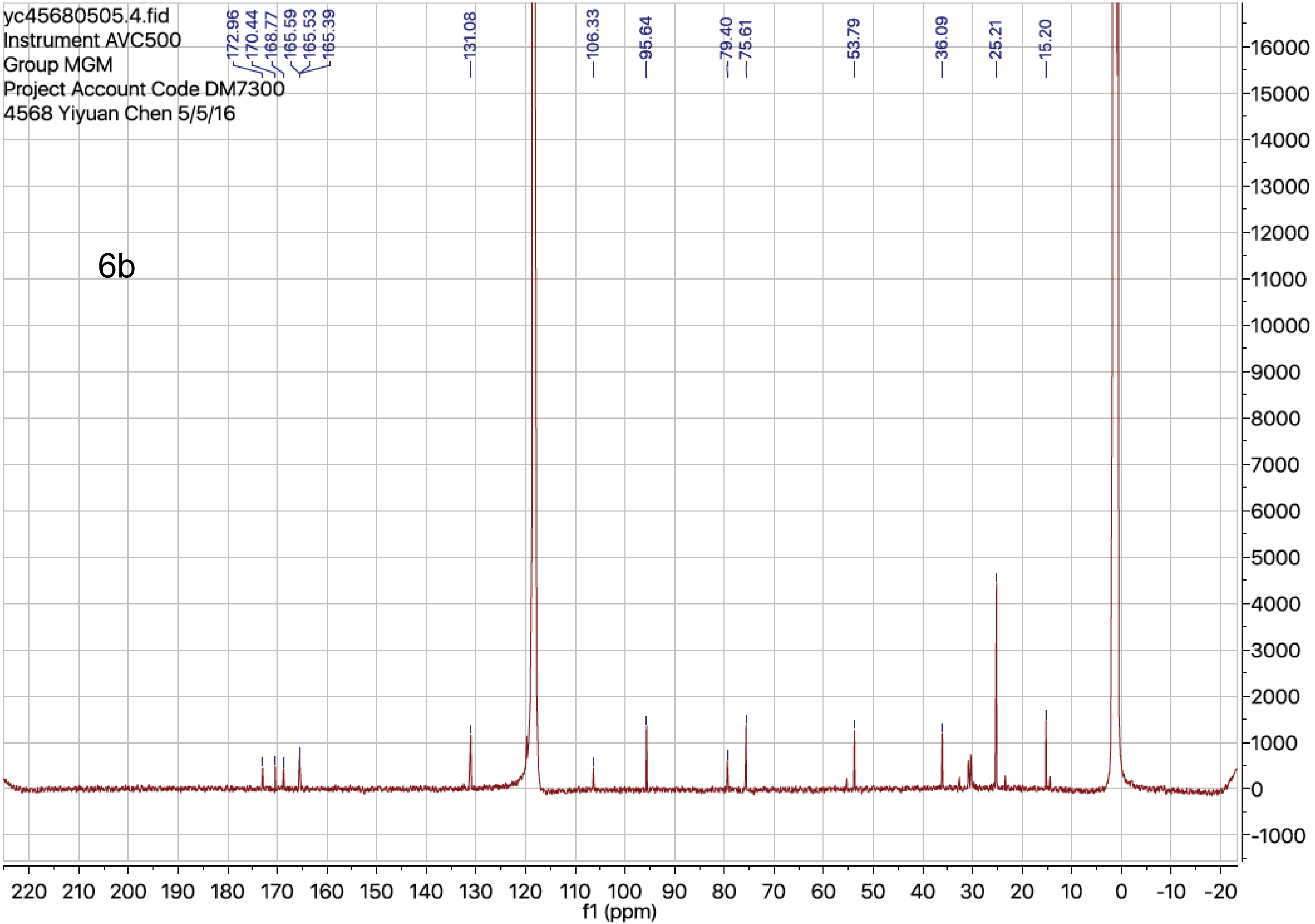

yc45680505.4.fid
Instrument AVC500
Group MGM
Project Account Code DM7300
4568 Yiyuan Chen 5/5/16
6b
172.96
170.44
168.77
165.59
165.53
165.39
131.08
106.33
95.64
79.40
75.61
53.79
36.09
25.21
15.20
f1 (ppm)

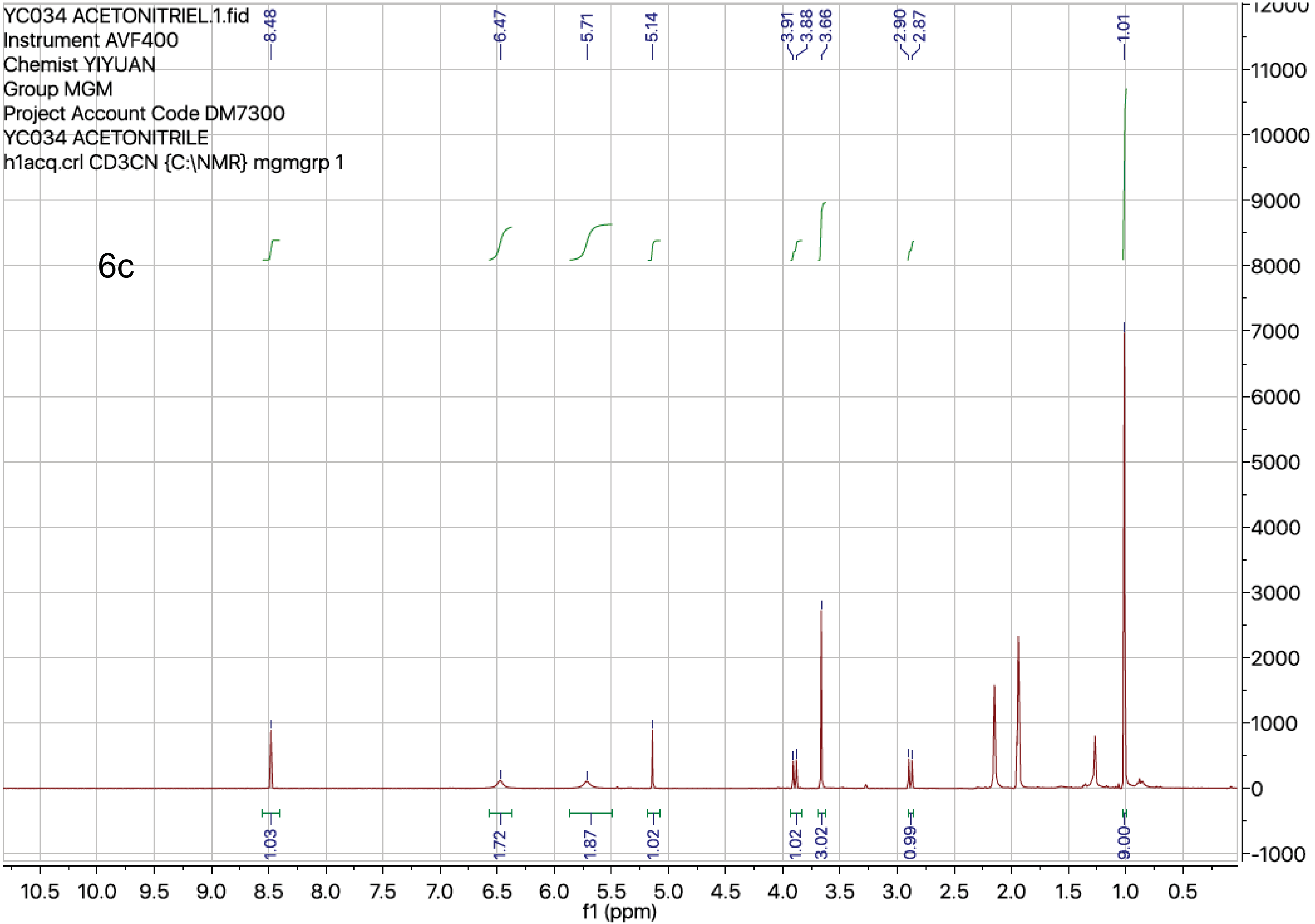
YC034 ACETONITRIEL.1.fid
Instrument AVF400
Chemist YIYUAN
Group MGM
Project Account Code DM7300
YC034 ACETONITRILE
h1acq.crl CD3CN {C:\NMR} mgmgrp 1
6c
8.48
6.47
5.71
5.14
3.91
3.88
3.66
2.90
2.87
1.01
1.03
1.72
1.87
1.02
1.02
3.02
0.99
9.00
f1 (ppm)

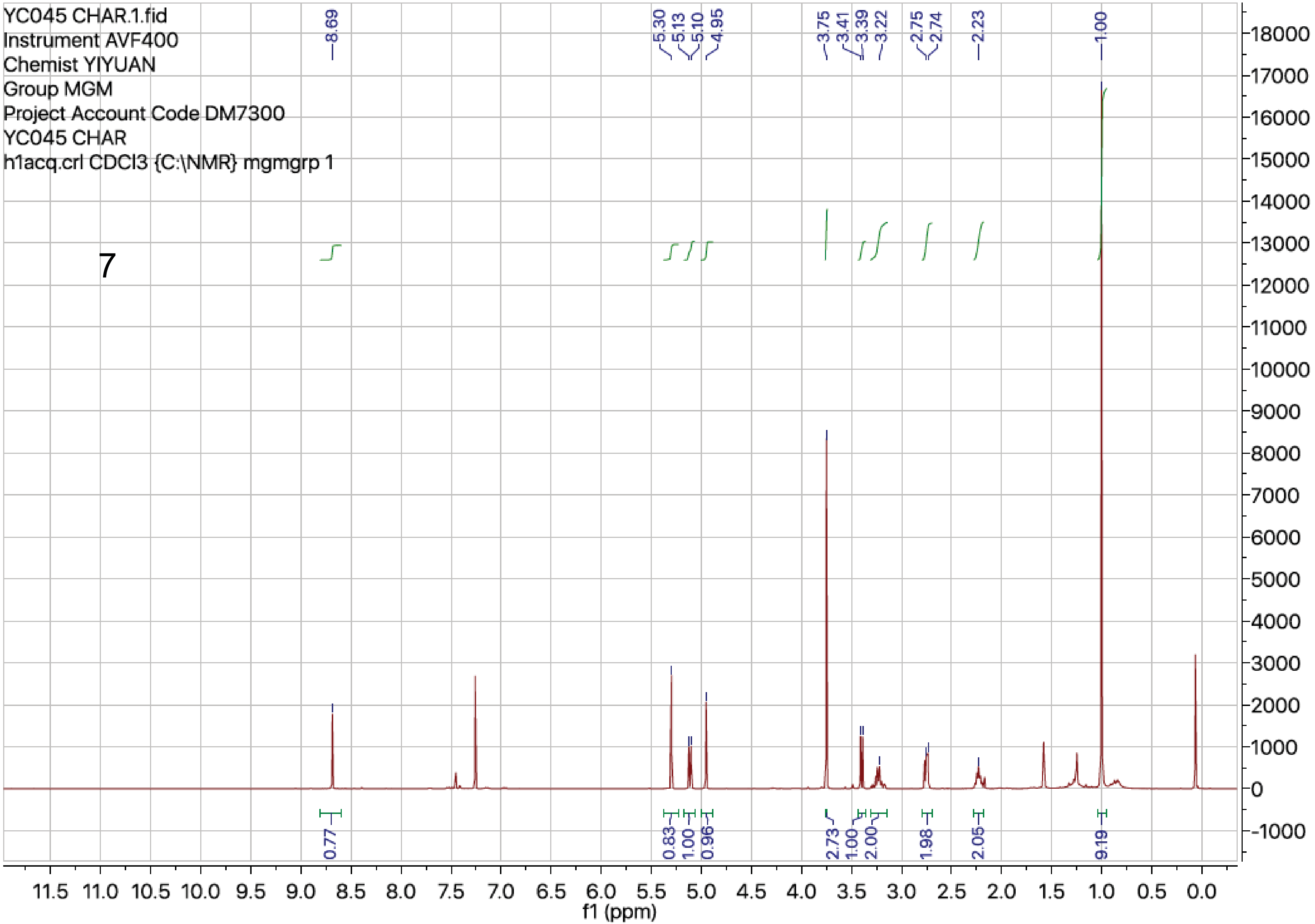

YC045 CHAR.1.fid
Instrument AVF400
Chemist YIYUAN
Group MGM
Project Account Code DM7300
YC045 CHAR
h1acq.crl CDCl3 {C:\NMR} mgmgrp 1
7
8.69
5.30
5.13
5.10
4.95
3.75
3.41
3.39
3.22
2.75
2.74
2.23
1.00
0.77
0.83
1.00
0.96
2.73
1.00
2.00
1.98
2.05
9.19
f1 (ppm)

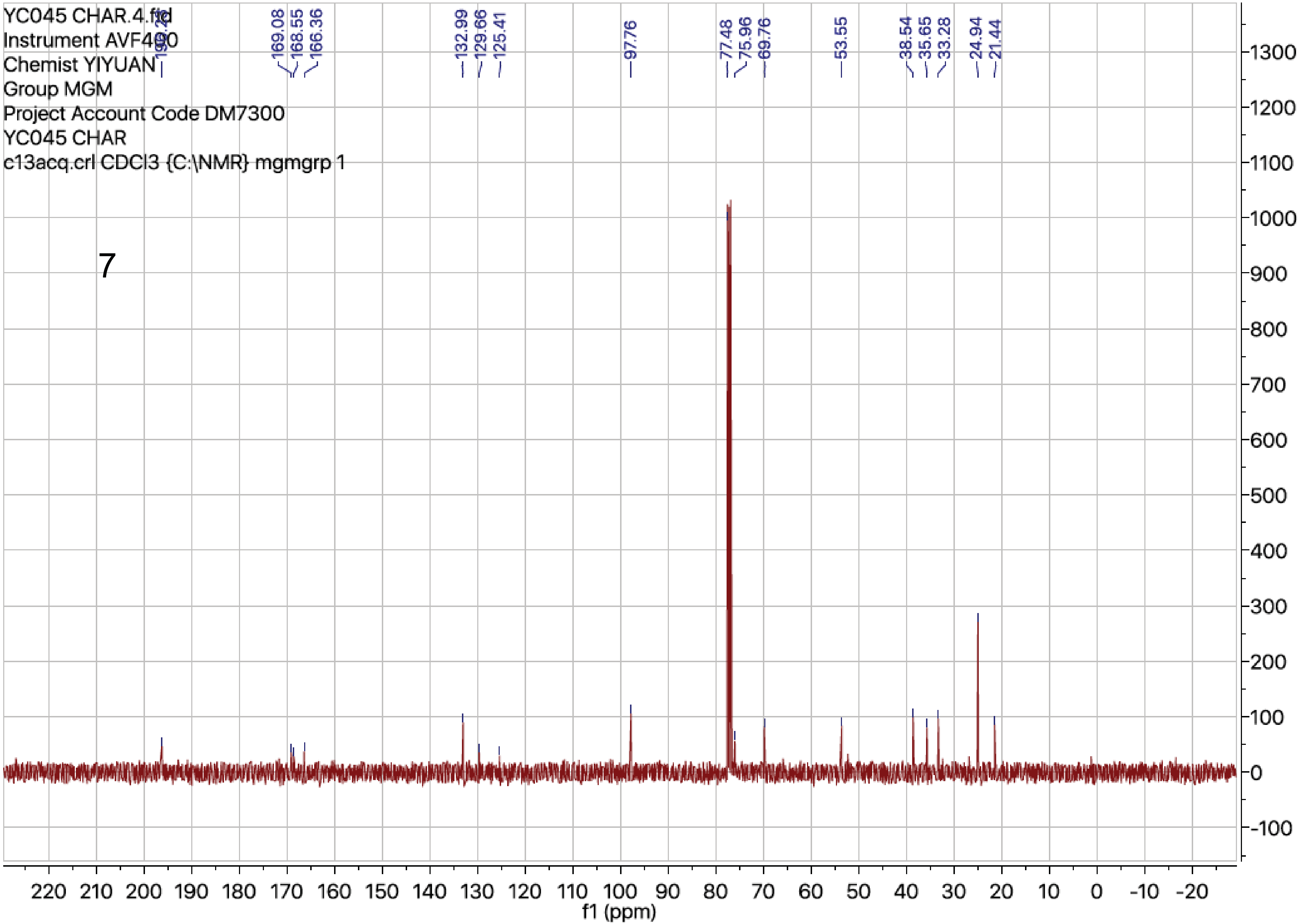
YC045 CHAR.4.fid
Instrument AVF400
Chemist YIYUAN
Group MGM
Project Account Code DM7300
YC045 CHAR
c13acq.crl CDCl3 {C:\NMR} mgmgrp 1
7
169.08
168.55
166.36
132.99
129.66
125.41
97.76
77.48
75.96
69.76
53.55
38.54
35.65
33.28
24.94
21.44
f1 (ppm)

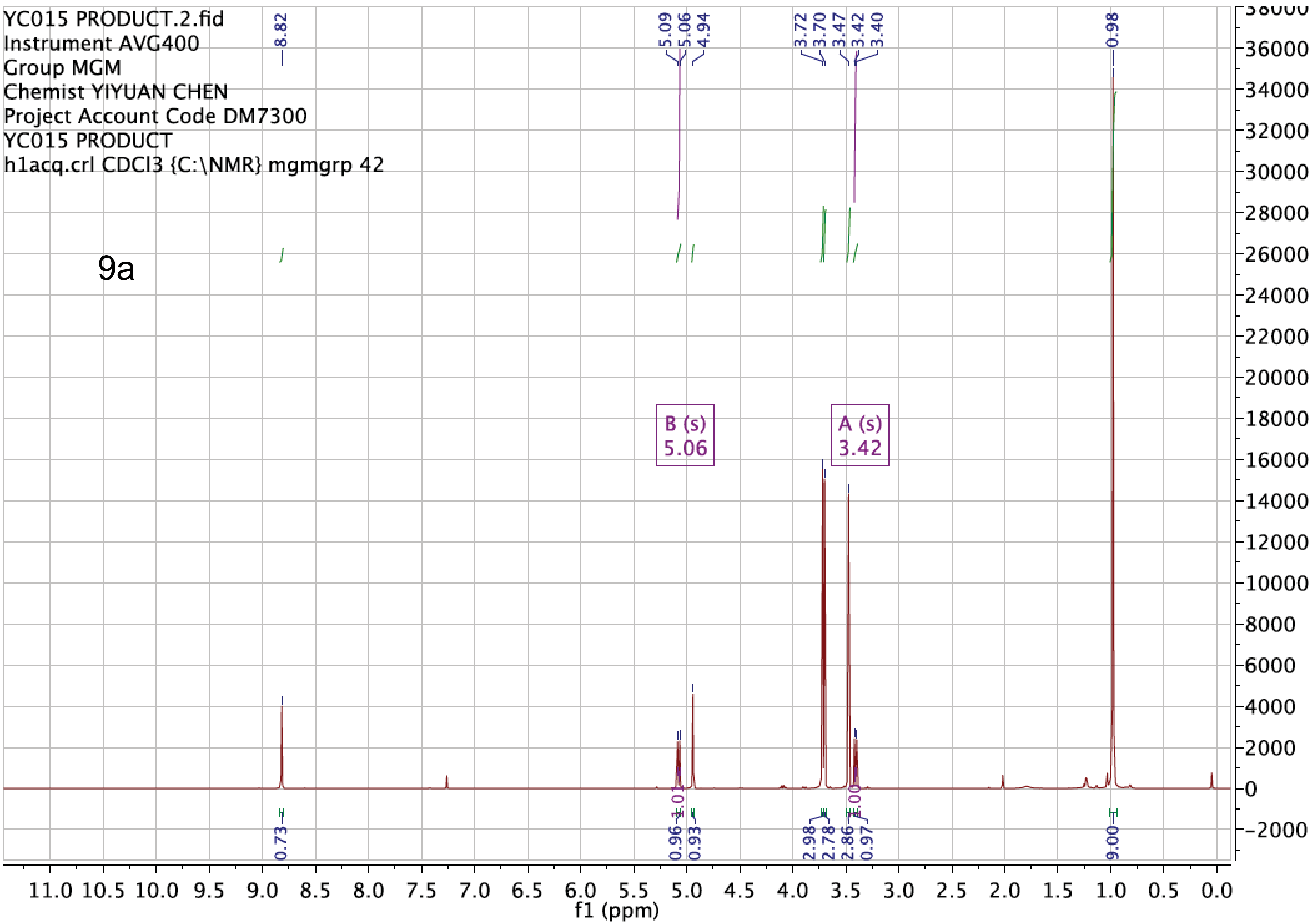
YC015 PRODUCT.2.fid
Instrument AVG400
Group MGM
Chemist YIYUAN CHEN
Project Account Code DM7300
YC015 PRODUCT
h1acq.crl CDCl3 {C:\NMR} mgmgrp 42
9a
8.82
5.09
5.06
4.94
3.72
3.70
3.47
3.42
3.40
0.98
B (s)
5.06
A (s)
3.42
0.73
0.96
1.01
0.93
2.98
2.78
2.86
1.00
0.97
9.00
f1 (ppm)

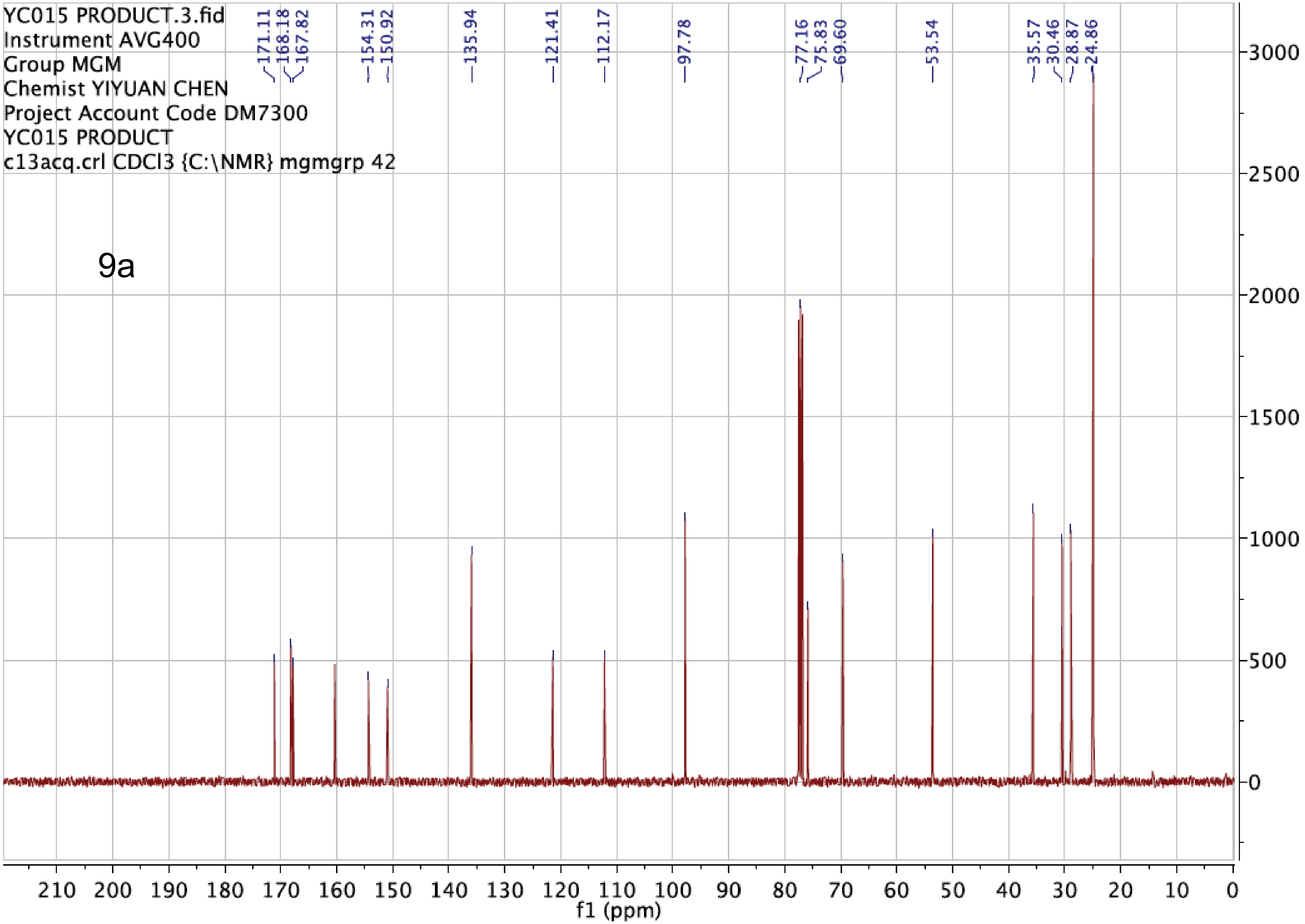
YC015 PRODUCT.3.fid
Instrument AVG400
Group MGM
Chemist YIYUAN CHEN
Project Account Code DM7300
YC015 PRODUCT
c13acq.crl CDCl3 {C:\NMR} mgmgrp 42
9a
171.11
168.18
167.82
154.31
150.92
135.94
121.41
112.17
97.78
77.16
75.83
69.60
53.54
35.57
30.46
28.87
24.86
f1 (ppm)

9a

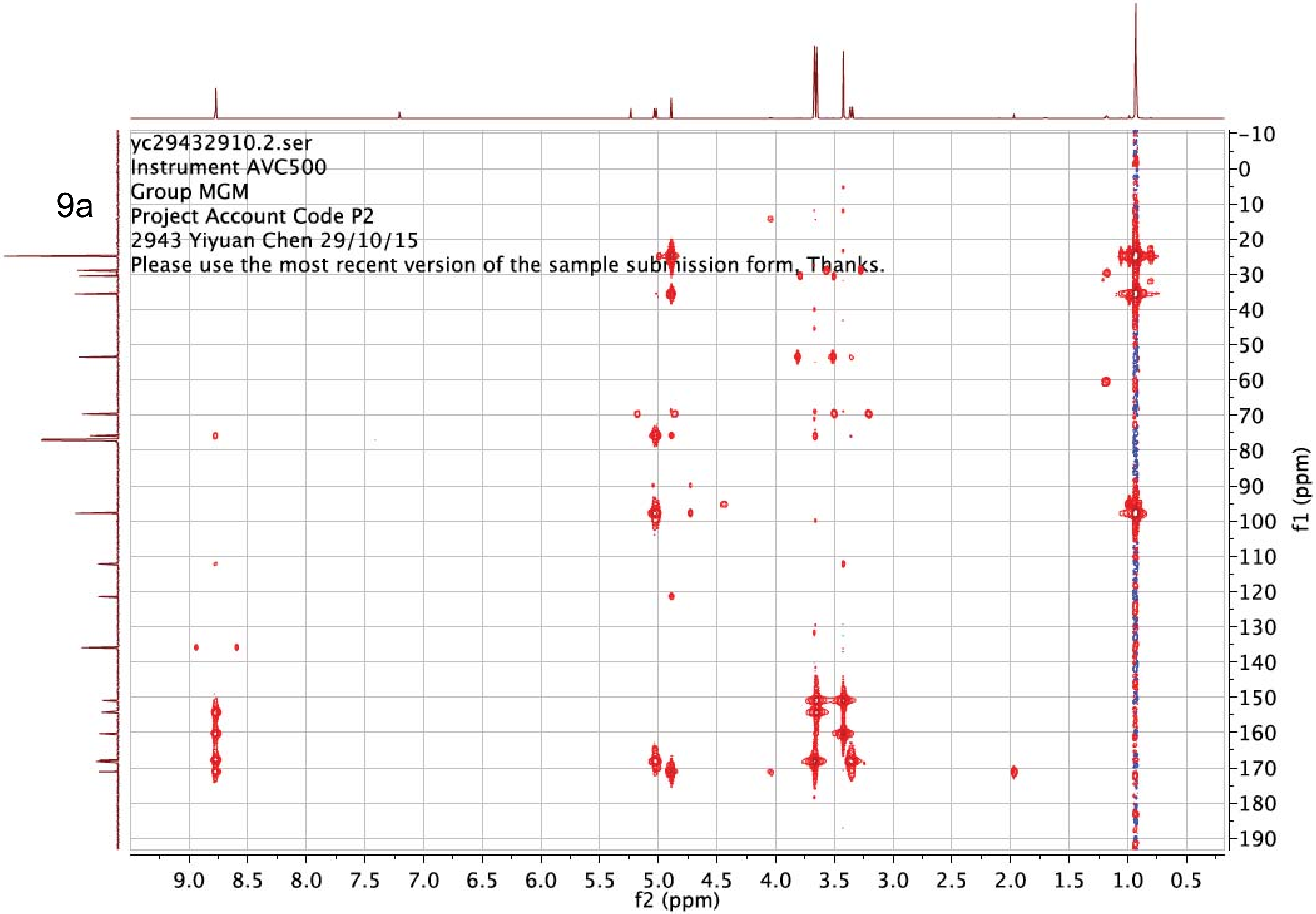

yc29432910.2.ser
Instrument AVC500
Group MGM
Project Account Code P2
2943 Yiyuan Chen 29/10/15
Please use the most recent version of the sample submission form. Thanks.
f2 (ppm)
f1 (ppm)

Instrument AVF400
Chemist YIYUAN
Group MGM
Project Account Code DM7300
YC070 MeOD
h1acq.crl MeOD {C:\NMR} mgmgrp 5

9b

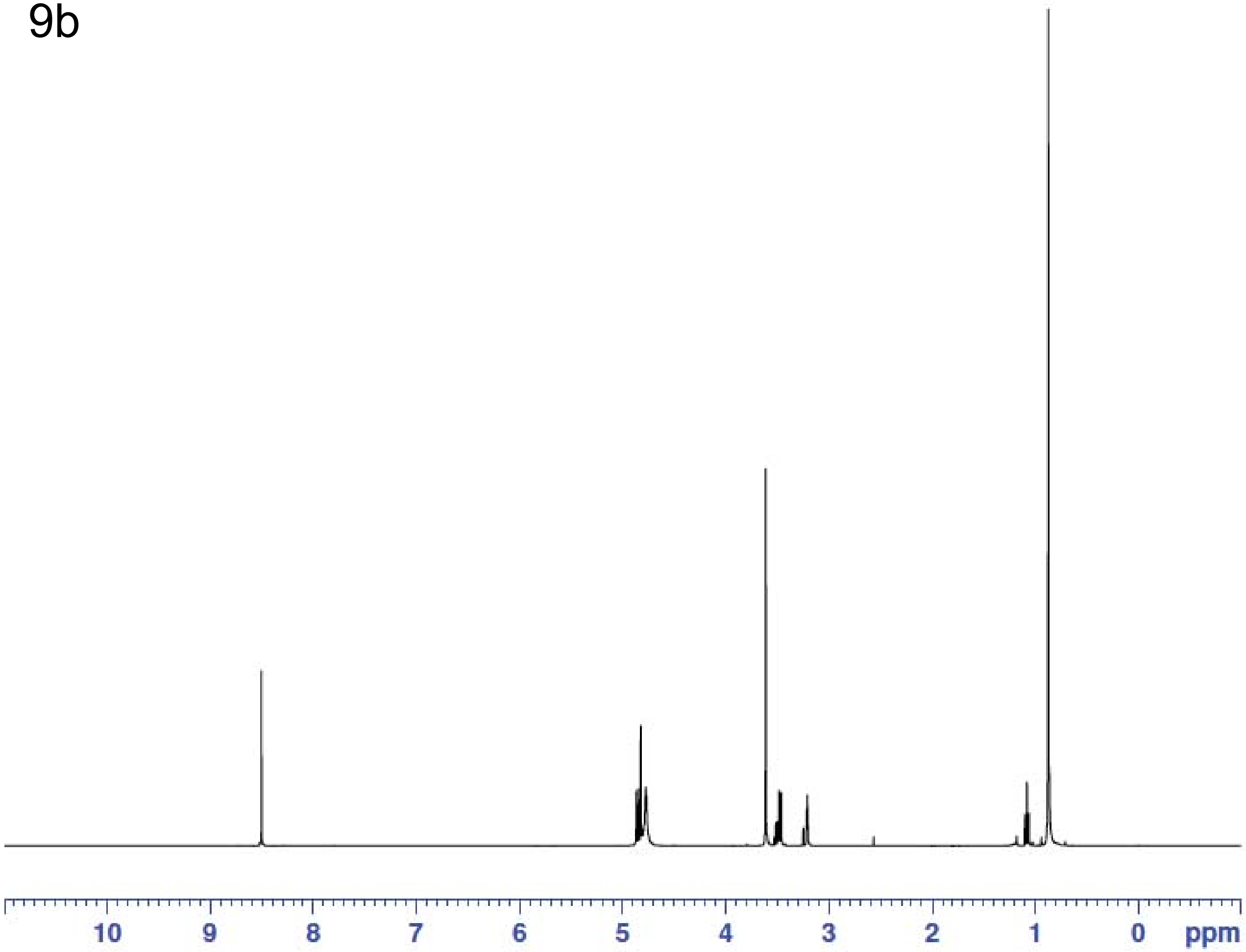

NMR@CHEM.OX

```
Current Data Parameters
NAME          YC070 MeOD
EXPNO                  1
PROCNO                 1

F2 - Acquisition Parameters
Date_           20160423
Time               12.15 h
INSTRUM           avf400
PROBHD   Z108618_0533 (
PULPROG             zg60
TD                 65536
SOLVENT             MeOD
NS                    16
DS                     2
SWH             8012.820 Hz
FIDRES          0.244532 Hz
AQ             4.0894465 sec
RG                 65.71
DW                62.400 usec
DE                  6.50 usec
TE                 295.0 K
D1            1.00000000 sec
TD0                    1
SFO1         400.2524015 MHz
NUC1                  1H
P1                 19.50 usec
PLW1         16.70000076 W

F2 - Processing parameters
SI                 32768
SF           400.2500484 MHz
WDW                   EM
SSB      0
LB                  0.30 Hz
GB       0
PC                  1.00
```

Instrument AVF400
Chemist YIYUAN
Group MGM
Project Account Code DM7300
YC070 MeOD
c13acq.crl MeOD {C:\NMR} mqmqrp 5

9b

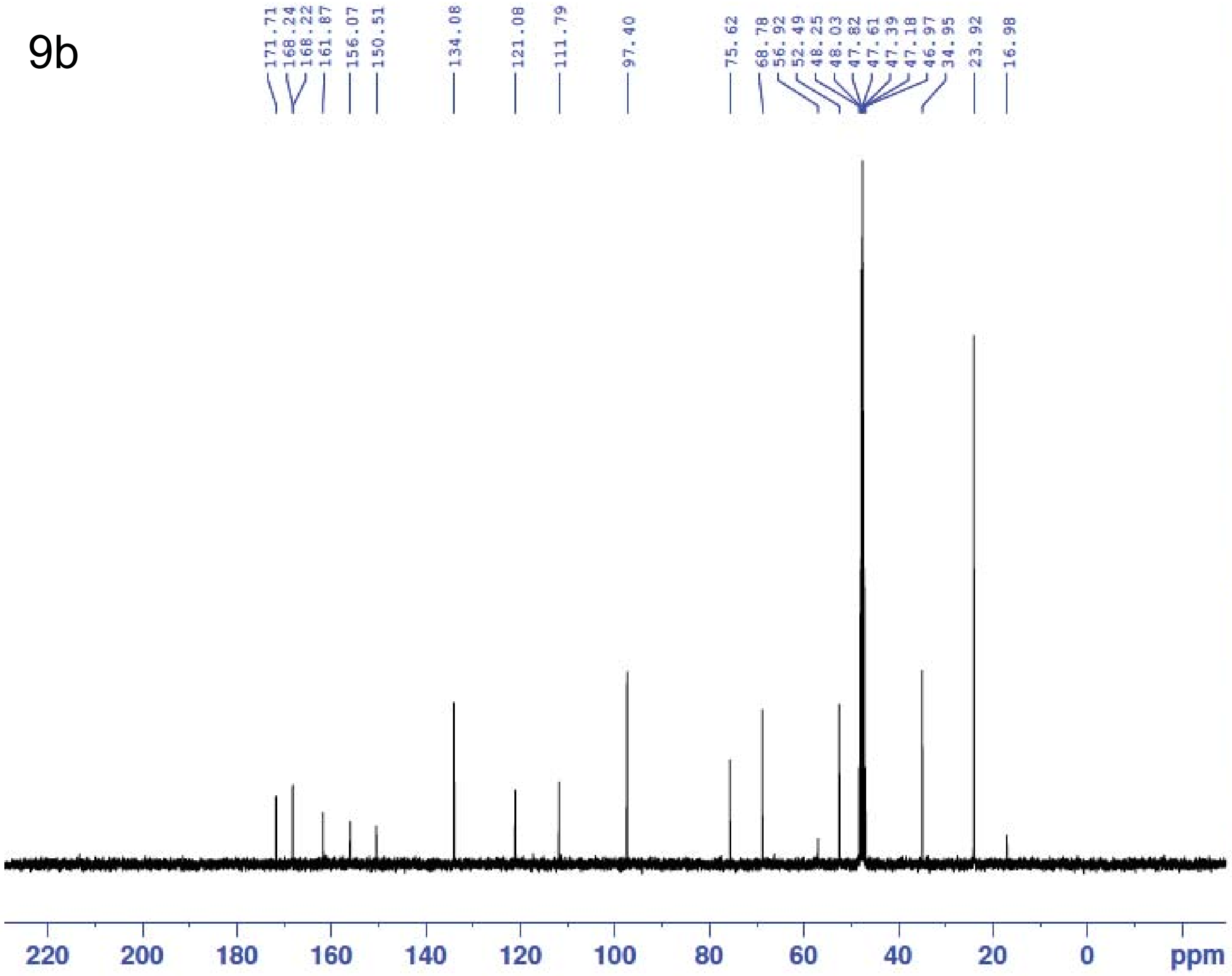

NMR@CHEM.OX

```
Current Data Parameters
NAME            YC070 MeOD
EXPNO                    4
PROCNO                   1

F2 - Acquisition Parameters
Date_             20160423
Time                 12.39 h
INSTRUM             avf400
PROBHD    Z108618_0533 (
PULPROG            zgpg30
TD                  32768
SOLVENT              MeOD
NS                    256
DS                      4
SWH             26041.666 Hz
FIDRES           1.589457 Hz
AQ              0.6291456 sec
RG                 205.43
DW                 19.200 usec
DE                   6.50 usec
TE                  295.7 K
D1             1.00000000 sec
D11            0.03000000 sec
TD0                     1
SFO1          100.6530068 MHz
NUC1                  13C
P1                   9.00 usec
PLW1          58.70000076 W
SFO2          400.2516010 MHz
NUC2                   1H
CPDPRG[2          waltz16
PCPD2               90.00 usec
PLW2          16.70000076 W
PLW12          0.78397000 W
PLW13          0.39432999 W

F2 - Processing parameters
SI                  32768
SF            100.6429430 MHz
WDW                    EM
SSB      0
LB                   1.00 Hz
GB       0
PC                   1.40
```

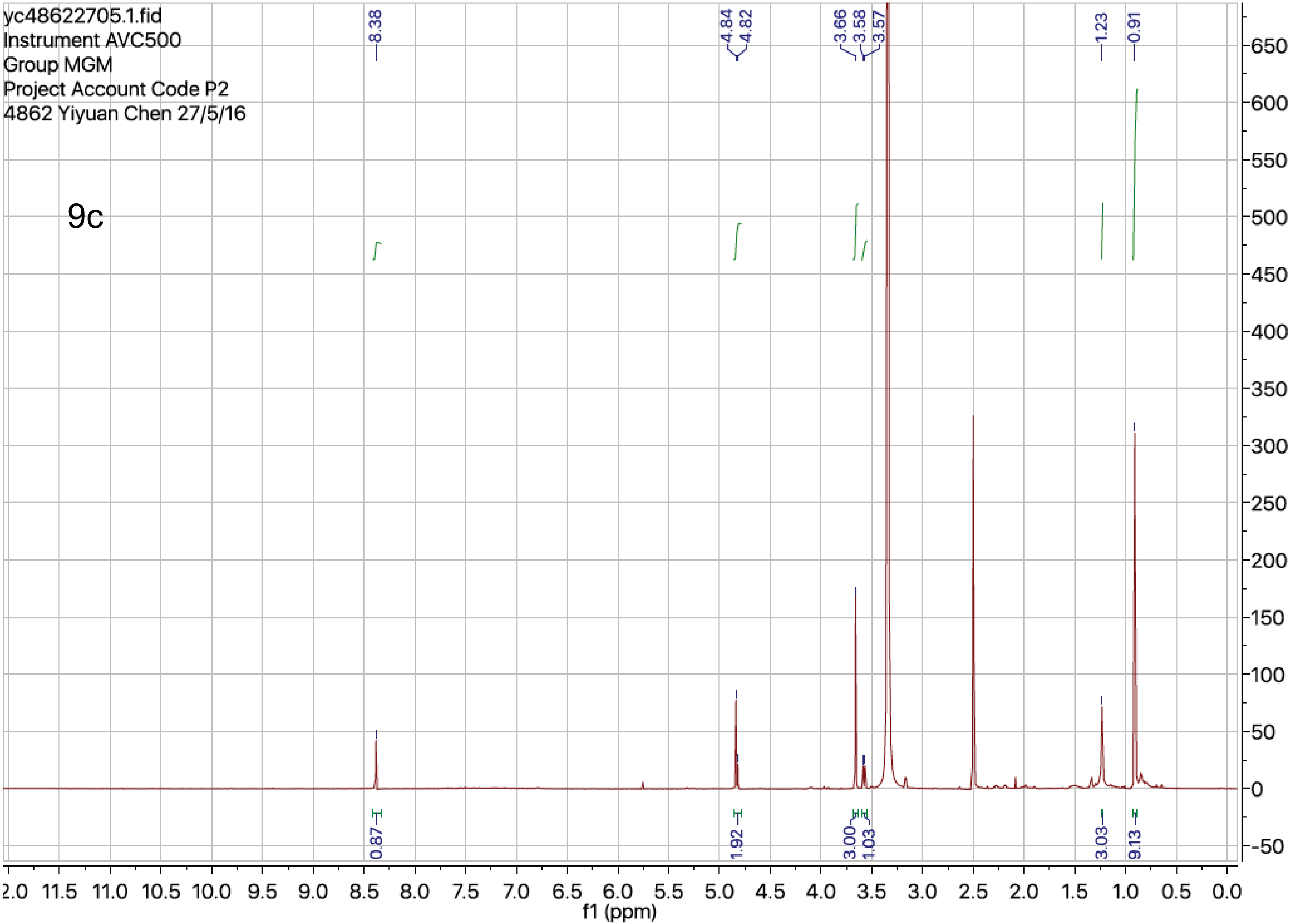

yc48622705.1.fid
Instrument AVC500
Group MGM
Project Account Code P2
4862 Yiyuan Chen 27/5/16
9c
8.38
4.84
4.82
3.66
3.58
3.57
1.23
0.91
0.87
1.92
3.00
1.03
3.03
9.13
f1 (ppm)

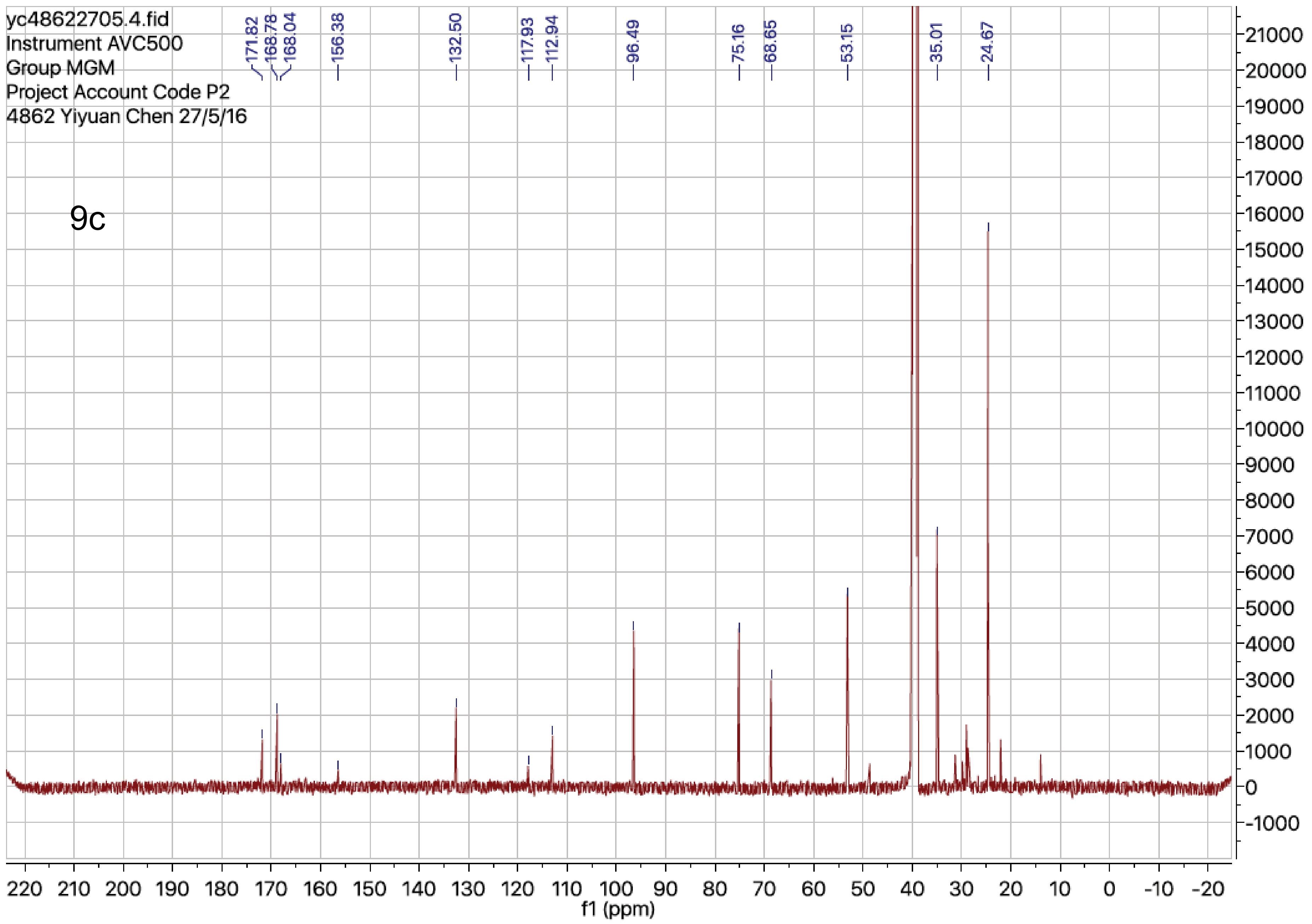
yc48622705.4.fid
Instrument AVC500
Group MGM
Project Account Code P2
4862 Yiyuan Chen 27/5/16
9c
171.82
168.78
168.04
156.38
132.50
117.93
112.94
96.49
75.16
68.65
53.15
35.01
24.67
f1 (ppm)

9c

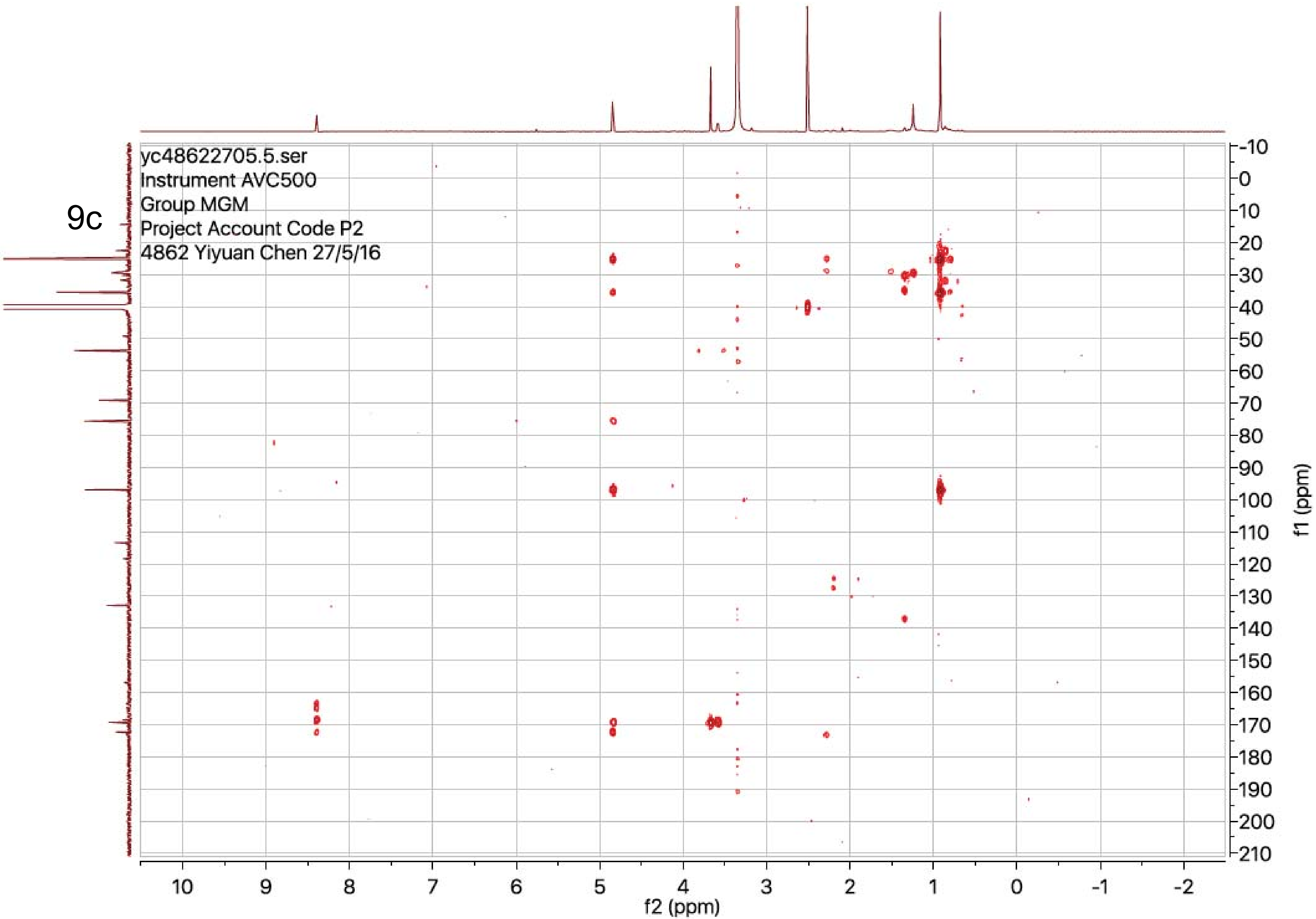
yc48622705.5.ser
Instrument AVC500
Group MGM
Project Account Code P2
4862 Yiyuan Chen 27/5/16
f2 (ppm)
f1 (ppm)

Instrument AVF400
Chemist YIYUAN
Group MGM
Project Account Code DM7300
YC071 AFTER COLUMN
h1acq.crl MeOD {C:\NMR} mgmgrp 1

NMR@CHEM.OX

9d

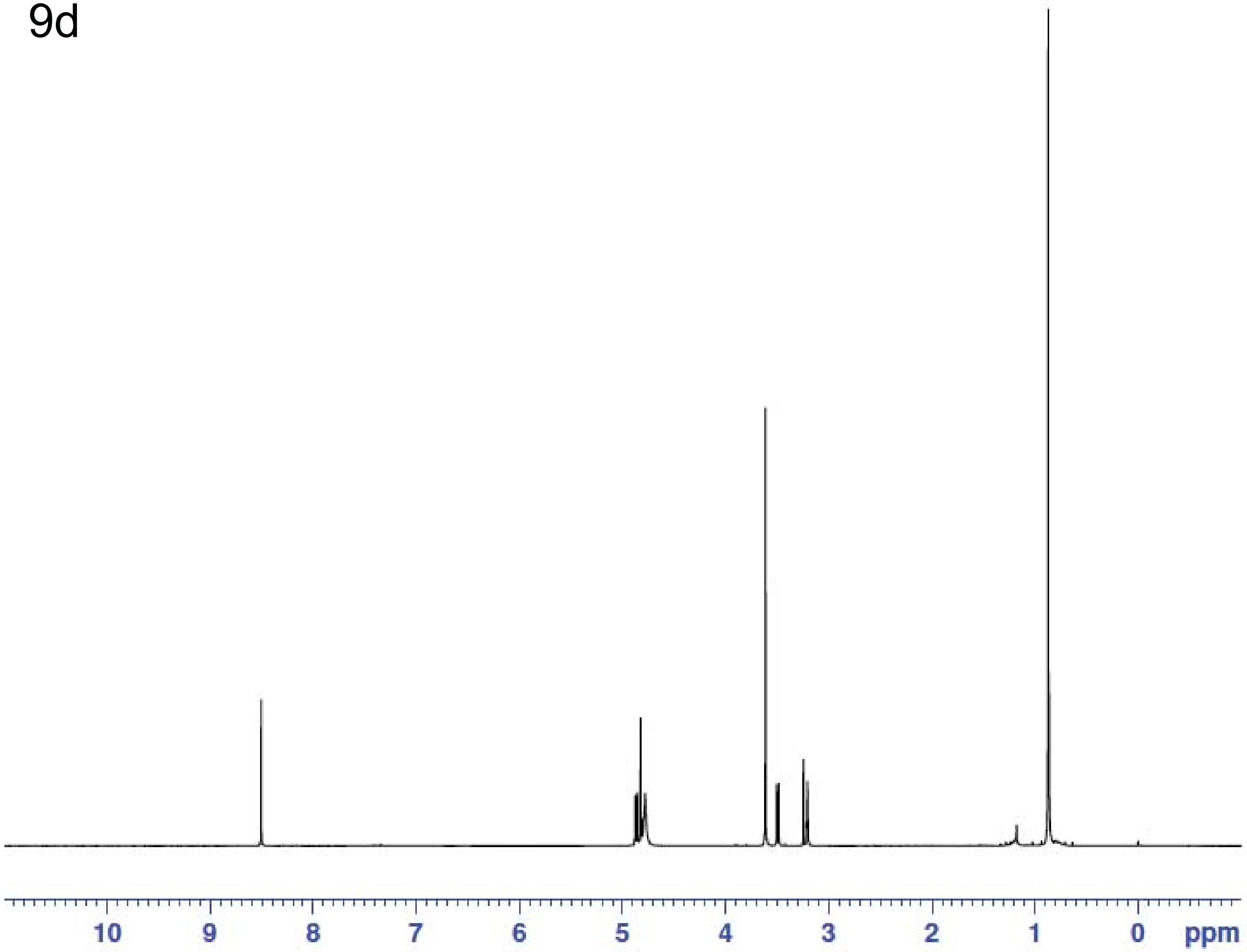

Current Data Parameters

NAME	YC071 Column
EXPNO	1
PROCNO	1

F2 - Acquisition Parameters

Date_	20160503
Time	12.27 h
INSTRUM	avf400
PROBHD	Z108618_0533 (
PULPROG	zg60
TD	65536
SOLVENT	MeOD
NS	16
DS	2
SWH	8012.820 Hz
FIDRES	0.244532 Hz
AQ	4.0894465 sec
RG	65.71
DW	62.400 usec
DE	6.50 usec
TE	294.4 K
D1	1.00000000 sec
TD0	1
SFO1	400.2524015 MHz
NUC1	1H
P1	19.50 usec
PLW1	16.70000076 W

F2 - Processing parameters

SI	32768
SF	400.2500482 MHz
WDW	EM
SSB	0
LB	0.30 Hz
GB	0
PC	1.00

Instrument AVF400
Chemist YIYUAN
Group MGM
Project Account Code DM7300
YC071 AFTER COLUMN
c13acq.crl MeOD {C:\NMR} mgmgrp 1

9d

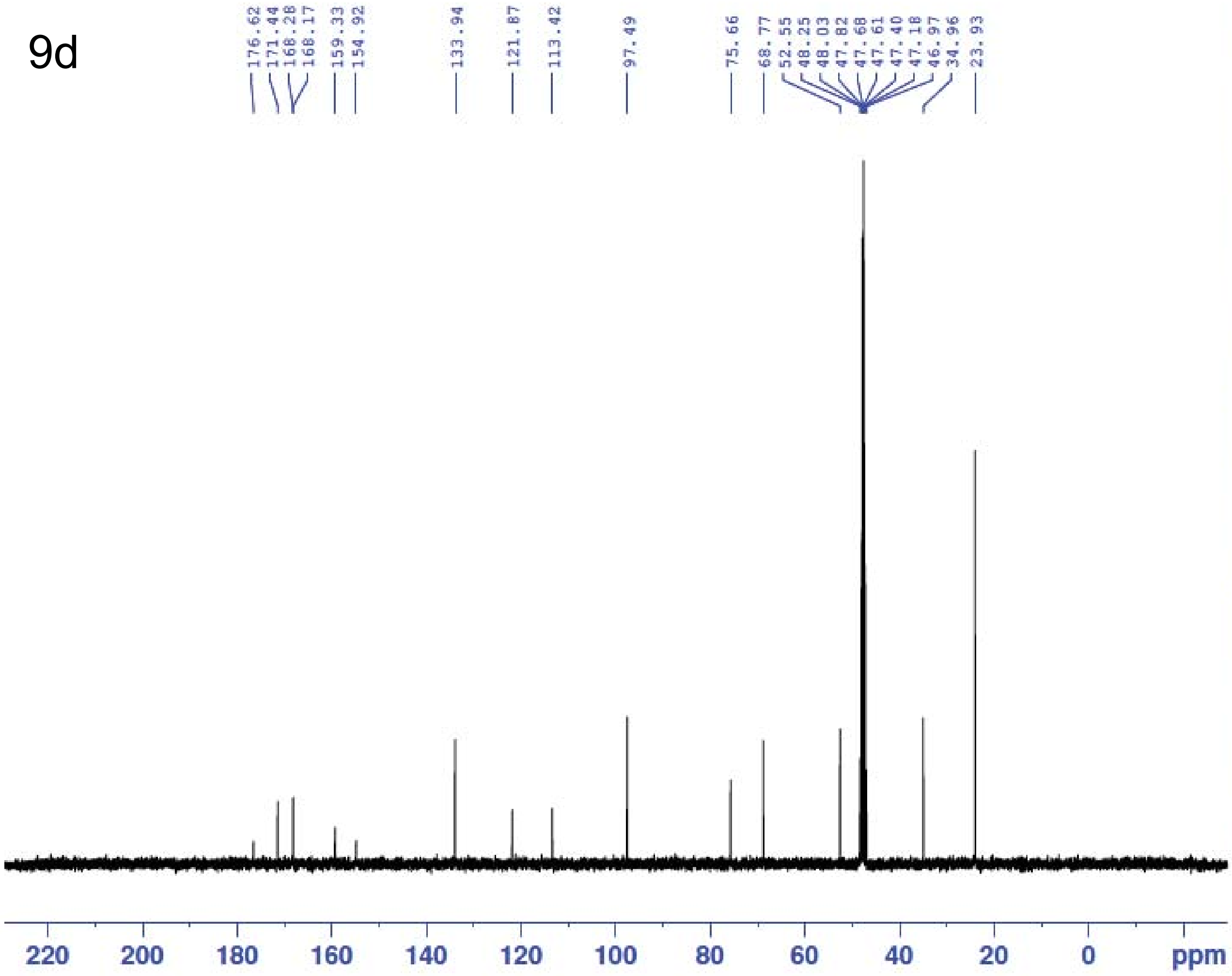

NMR@CHEM.OX

```
Current Data Parameters
NAME          YC071 Column
EXPNO                    4
PROCNO                   1

F2 - Acquisition Parameters
Date_             20160503
Time                 12.51 h
INSTRUM             avf400
PROBHD    Z108618_0533 (
PULPROG            zgpg30
TD                   32768
SOLVENT               MeOD
NS                     256
DS                       4
SWH             26041.666 Hz
FIDRES           1.589457 Hz
AQ              0.6291456 sec
RG                  205.43
DW                  19.200 usec
DE                    6.50 usec
TE                   295.1 K
D1             1.00000000 sec
D11            0.03000000 sec
TD0                      1
SFO1          100.6530068 MHz
NUC1                   13C
P1                    9.00 usec
PLW1          58.70000076 W
SFO2          400.2516010 MHz
NUC2                    1H
CPDPRG[2          waltz16
PCPD2                90.00 usec
PLW2          16.70000076 W
PLW12          0.78397000 W
PLW13          0.39432999 W

F2 - Processing parameters
SI                   32768
SF            100.6429430 MHz
WDW                     EM
SSB      0
LB                    1.00 Hz
GB       0
PC                    1.40
```

Instrument AVF400
Chemist YIYUAN CHEN
Group MGM
Project Account Code DM7300
YC030 CHARACT CDCl3
h1acq.crl CDCl3 {C:\NMR} mgmgrp 30

9e

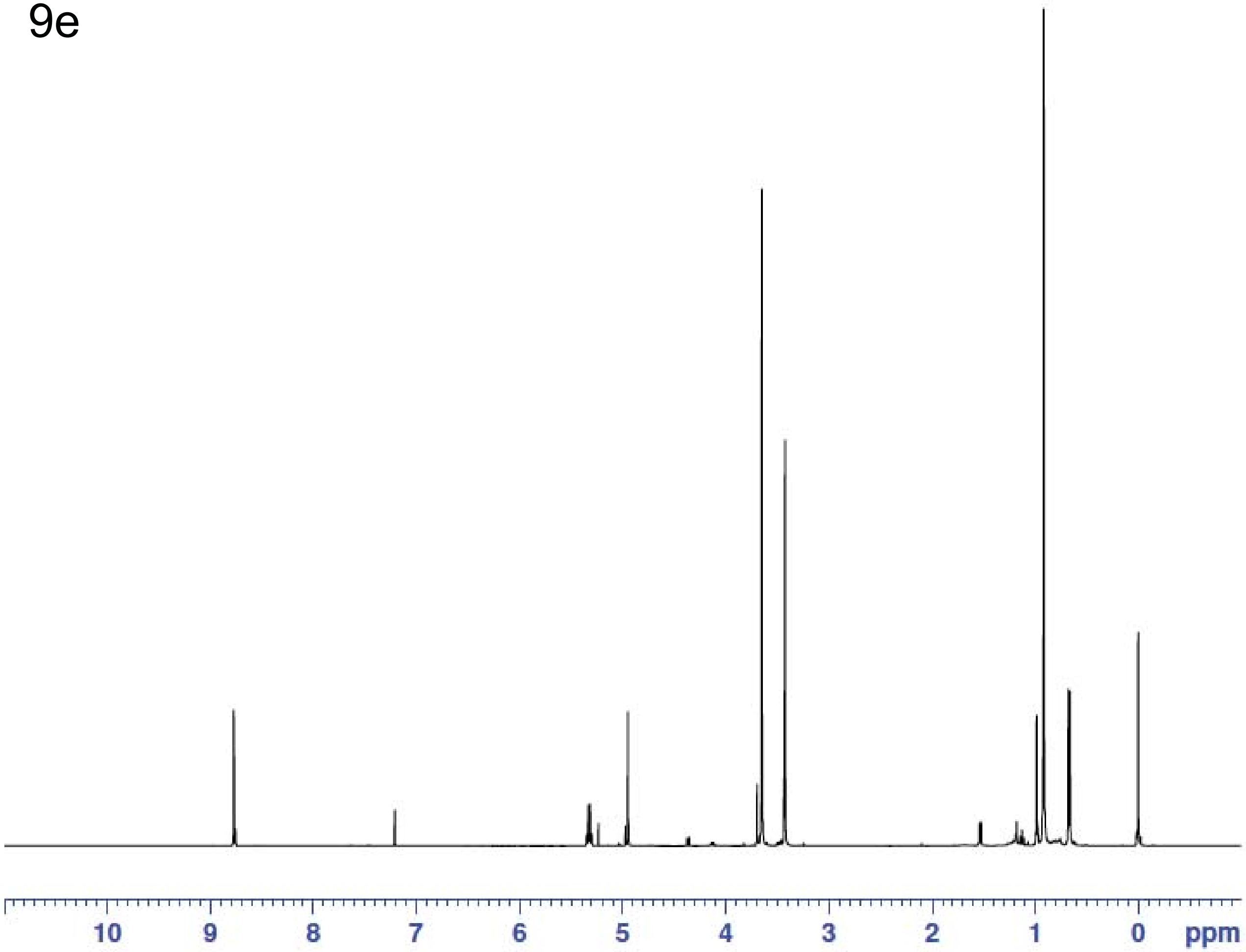

NMR@CHEM.OX

Current Data Parameters

NAME	YC030 CHAR
EXPNO	1
PROCNO	1

F2 - Acquisition Parameters

Date_	20160505
Time	7.04 h
INSTRUM	avf400
PROBHD	Z108618_0533 (
PULPROG	zg60
TD	65536
SOLVENT	CDCl3
NS	16
DS	2
SWH	8012.820 Hz
FIDRES	0.244532 Hz
AQ	4.0894465 sec
RG	39.49
DW	62.400 usec
DE	6.50 usec
TE	294.9 K
D1	1.00000000 sec
TD0	1
SFO1	400.2524015 MHz
NUC1	1H
P1	19.50 usec
PLW1	16.70000076 W

F2 - Processing parameters

SI	32768
SF	400.2500300 MHz
WDW	EM
SSB	0
LB	0.30 Hz
GB	0
PC	1.00

Instrument AVF400
Chemist YIYUAN CHEN
Group MGM
Project Account Code DM7300
YC030 CHARACT CDCl3
c13acq.crl CDCl3 {C:\NMR} mgmgrp 30

NMR@CHEM.OX

9e

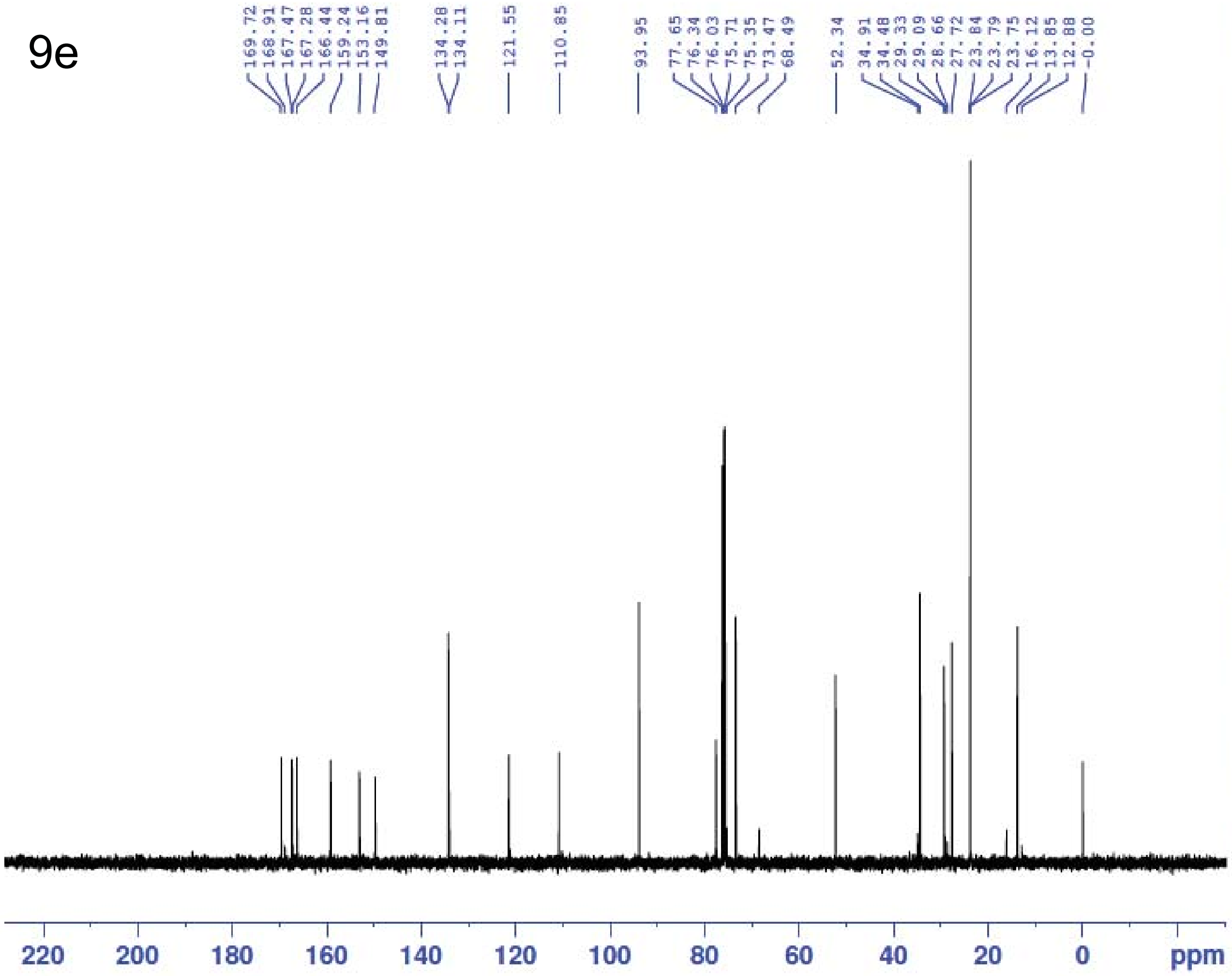

Current Data Parameters
NAME YC030 CHAR
EXPNO 4
PROCNO 1

F2 - Acquisition Parameters
Date_ 20160505
Time 7.27 h
INSTRUM avf400
PROBHD Z108618_0533 (
PULPROG zgpg30
TD 32768
SOLVENT CDCl3
NS 256
DS 4
SWH 26041.666 Hz
FIDRES 1.589457 Hz
AQ 0.6291456 sec
RG 205.43
DW 19.200 usec
DE 6.50 usec
TE 295.5 K
D1 1.00000000 sec
D11 0.03000000 sec
TD0 1
SFO1 100.6530068 MHz
NUC1 13C
P1 9.00 usec
PLW1 58.70000076 W
SFO2 400.2516010 MHz
NUC2 1H
CPDPRG[2 waltz16
PCPD2 90.00 usec
PLW2 16.70000076 W
PLW12 0.78397000 W
PLW13 0.39432999 W

F2 - Processing parameters
SI 32768
SF 100.6430464 MHz
WDW EM
SSB 0
LB 1.00 Hz
GB 0
PC 1.40

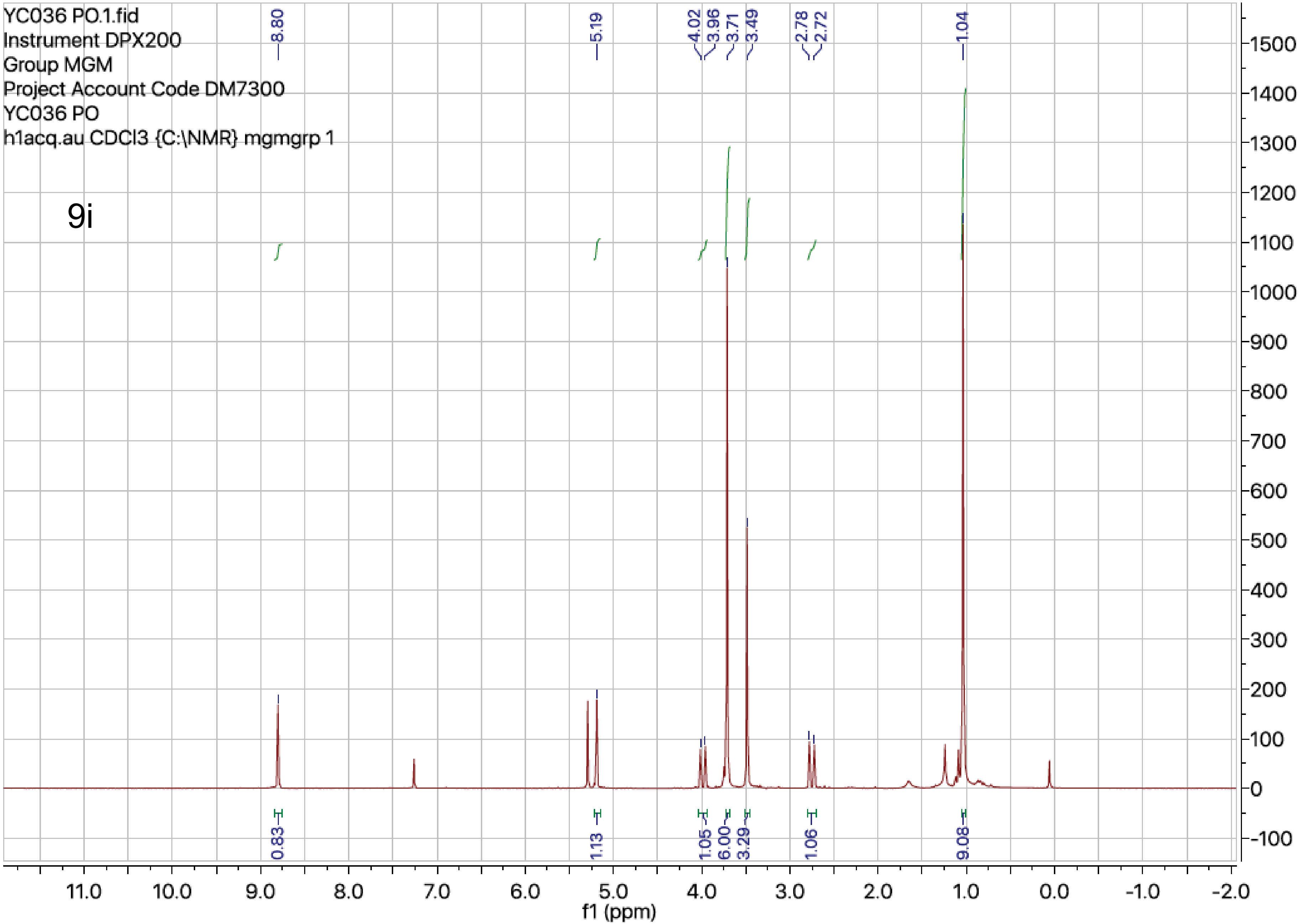

YC036 PO.1.fid
Instrument DPX200
Group MGM
Project Account Code DM7300
YC036 PO
h1acq.au CDCl3 {C:\NMR} mgmgrp 1
9i
8.80
5.19
4.02
3.96
3.71
3.49
2.78
2.72
1.04
0.83
1.13
1.05
6.00
3.29
1.06
9.08
f1 (ppm)

Instrument AVF400
Chemist YIYUAN CHEN
Group MGM
Project Account Code DM7300
YC033 BIO CHAR
c13acq.crl CDCl3 {C:\NMR} mgmgrp 33

9i

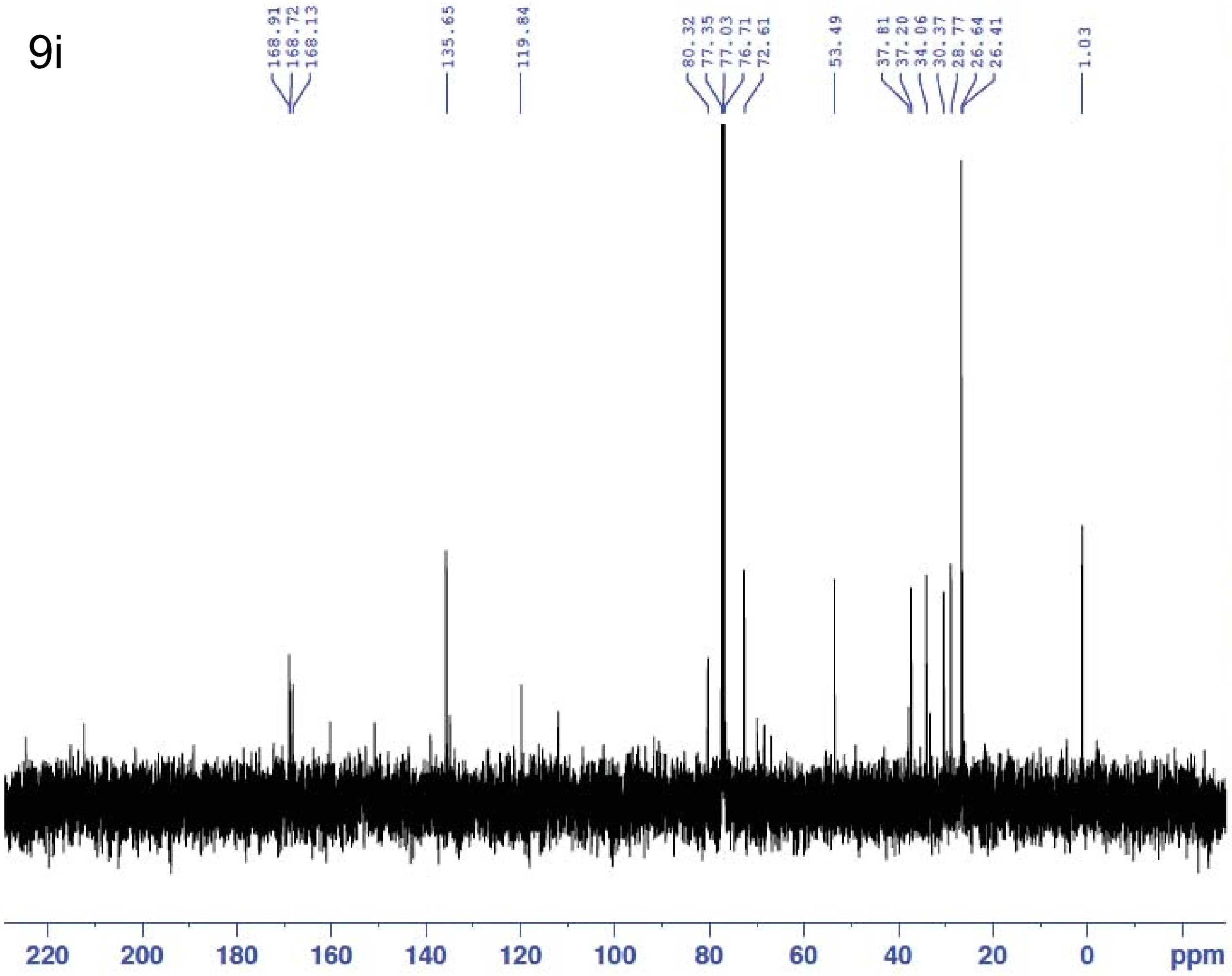

NMR@CHEM.OX

```
Current Data Parameters
NAME        YC033 BIO CHAR
EXPNO                    4
PROCNO                   1

F2 - Acquisition Parameters
Date_             20160505
Time                  6.54 h
INSTRUM             avf400
PROBHD    Z108618_0533 (
PULPROG            zgpg30
TD                   32768
SOLVENT              CDCl3
NS                     256
DS                       4
SWH              26041.666 Hz
FIDRES            1.589457 Hz
AQ               0.6291456 sec
RG                  205.43
DW                  19.200 usec
DE                    6.50 usec
TE                   295.5 K
D1              1.00000000 sec
D11             0.03000000 sec
TD0                      1
SFO1           100.6530068 MHz
NUC1                   13C
P1                    9.00 usec
PLW1           58.70000076 W
SFO2           400.2516010 MHz
NUC2                    1H
CPDPRG[2           waltz16
PCPD2                90.00 usec
PLW2           16.70000076 W
PLW12           0.78397000 W
PLW13           0.39432999 W

F2 - Processing parameters
SI                   32768
SF             100.6429430 MHz
WDW                     EM
SSB       0
LB                    1.00 Hz
GB        0
PC                    1.40
```

9j

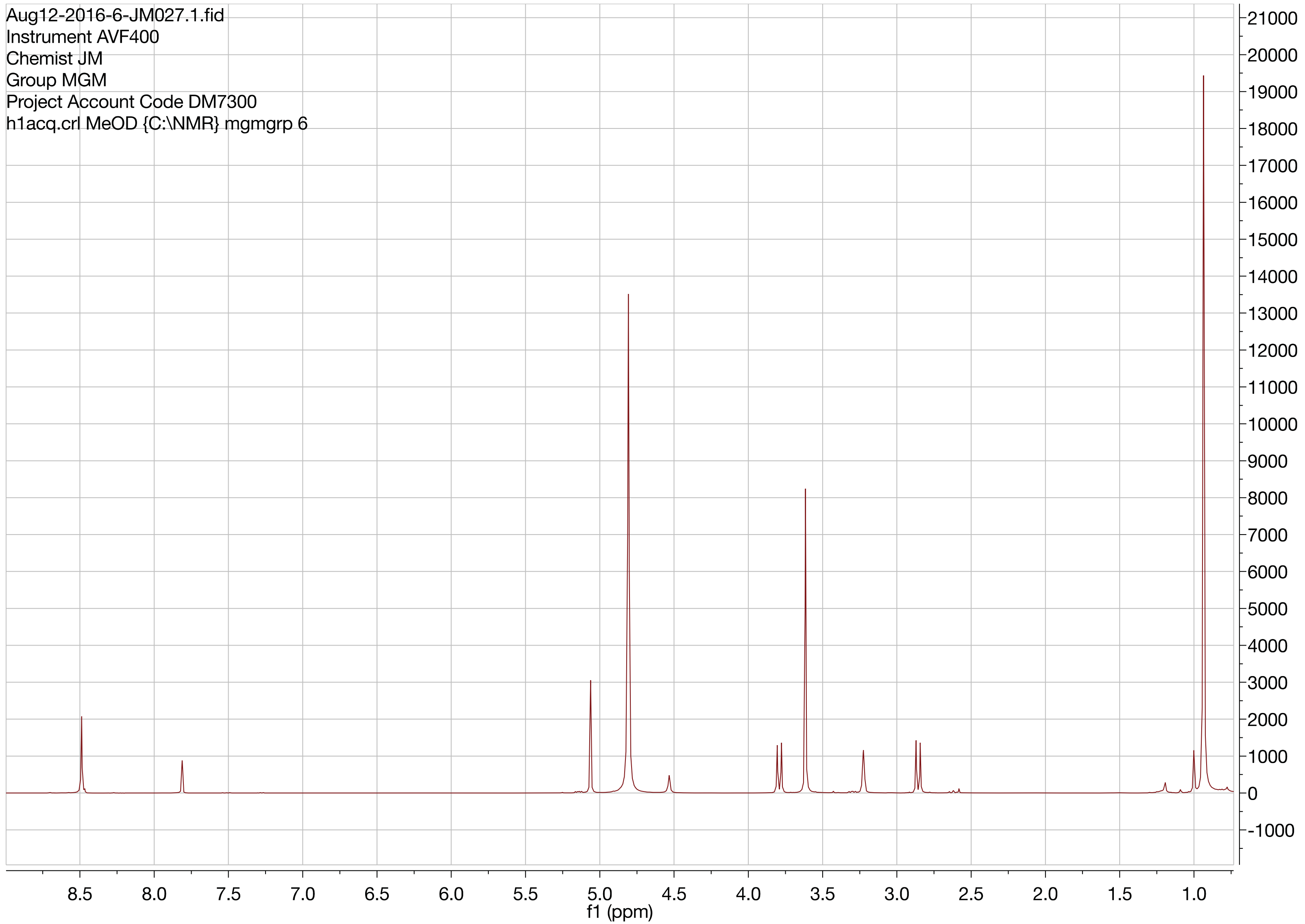

Aug12-2016-6-JM027.1.fid
Instrument AVF400
Chemist JM
Group MGM
Project Account Code DM7300
h1acq.crl MeOD {C:\NMR} mgmgrp 6
21000
20000
19000
18000
17000
16000
15000
14000
13000
12000
11000
10000
9000
8000
7000
6000
5000
4000
3000
2000
1000
0
-1000
8.5
8.0
7.5
7.0
6.5
6.0
5.5
5.0
4.5
4.0
3.5
3.0
2.5
2.0
1.5
1.0
f1 (ppm)

9j

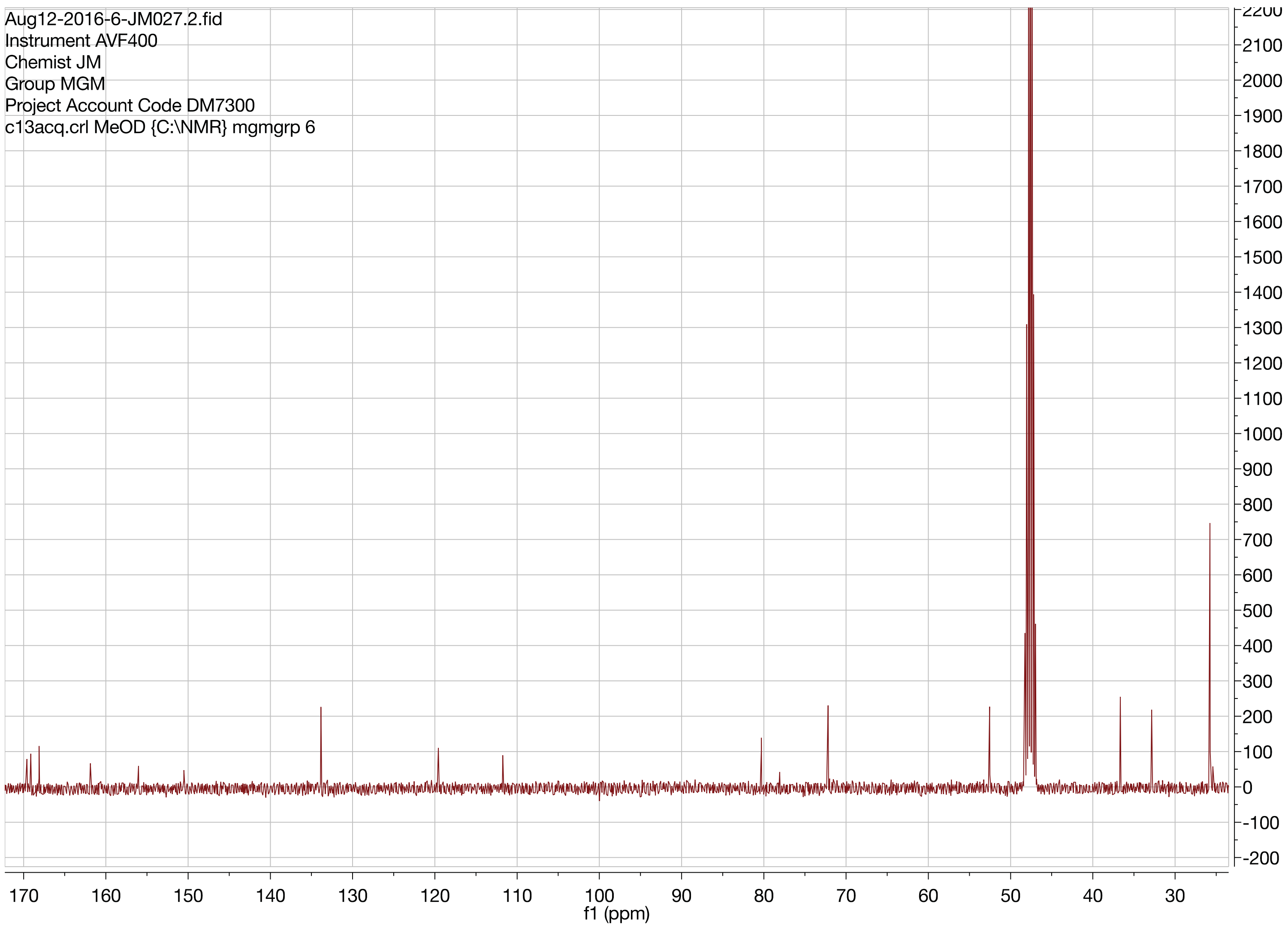
Aug12-2016-6-JM027.2.fid
Instrument AVF400
Chemist JM
Group MGM
Project Account Code DM7300
c13acq.crl MeOD {C:\NMR} mgmgrp 6
2200
2100
2000
1900
1800
1700
1600
1500
1400
1300
1200
1100
1000
900
800
700
600
500
400
300
200
100
0
-100
-200
170
160
150
140
130
120
110
100
90
80
70
60
50
40
30
f1 (ppm)

9j

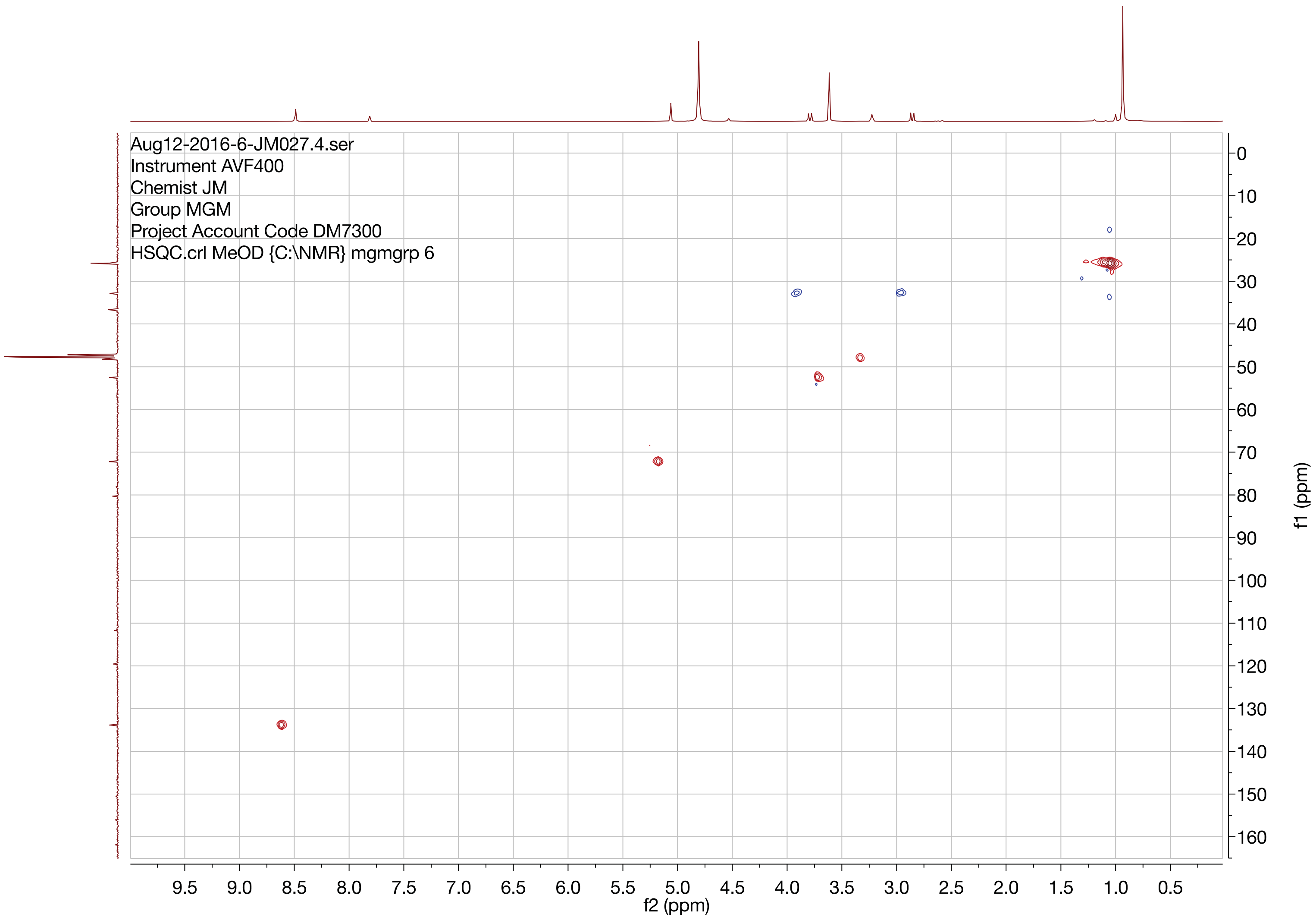

Aug12-2016-6-JM027.4.ser
Instrument AVF400
Chemist JM
Group MGM
Project Account Code DM7300
HSQC.crl MeOD {C:\NMR} mgmgrp 6
9.5
9.0
8.5
8.0
7.5
7.0
6.5
6.0
5.5
5.0
4.5
4.0
3.5
3.0
2.5
2.0
1.5
1.0
0.5
f2 (ppm)
0
10
20
30
40
50
60
70
80
90
100
110
120
130
140
150
160
f1 (ppm)

HMBC

9j

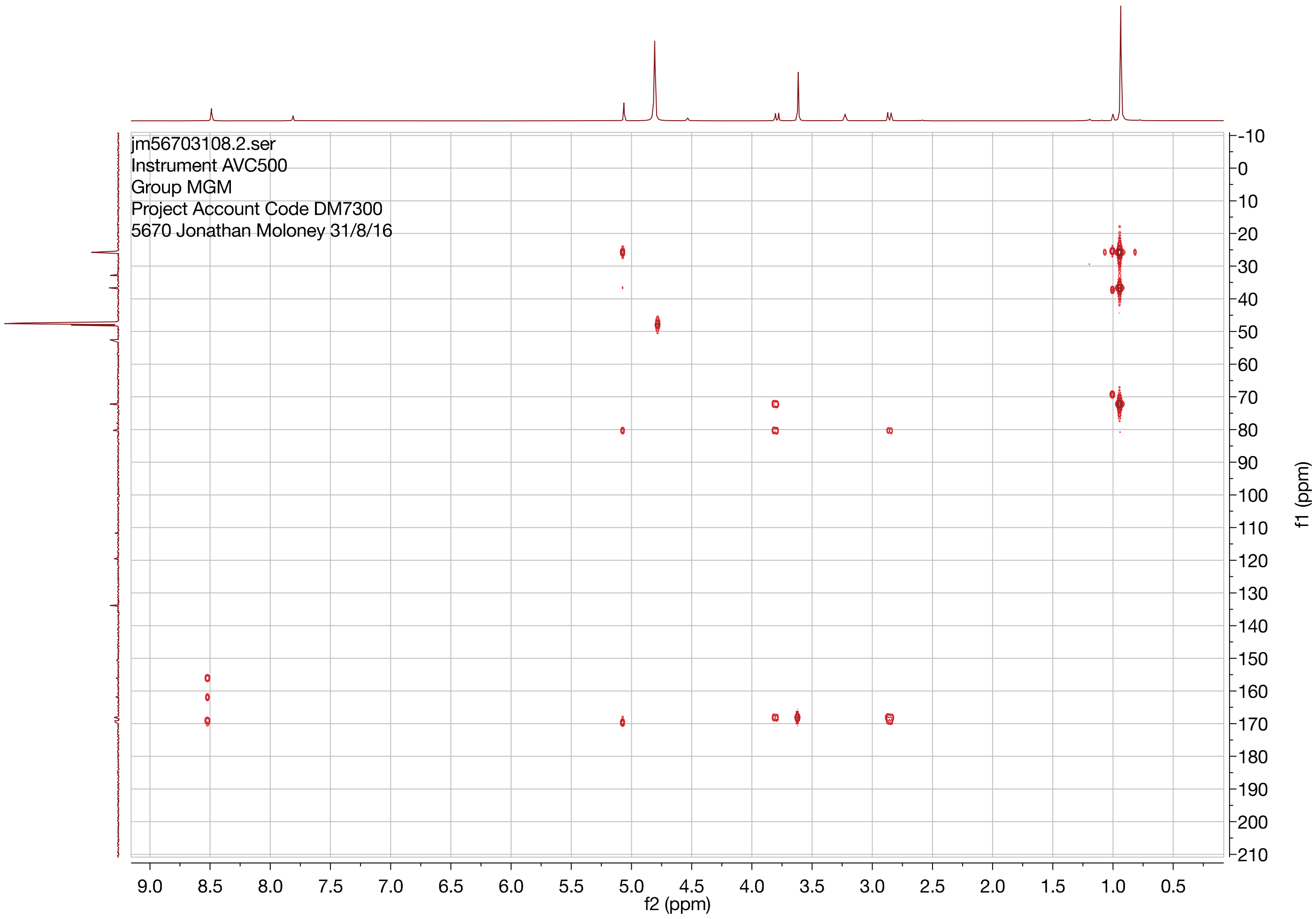
jm56703108.2.ser
Instrument AVC500
Group MGM
Project Account Code DM7300
5670 Jonathan Moloney 31/8/16
f2 (ppm)
f1 (ppm)

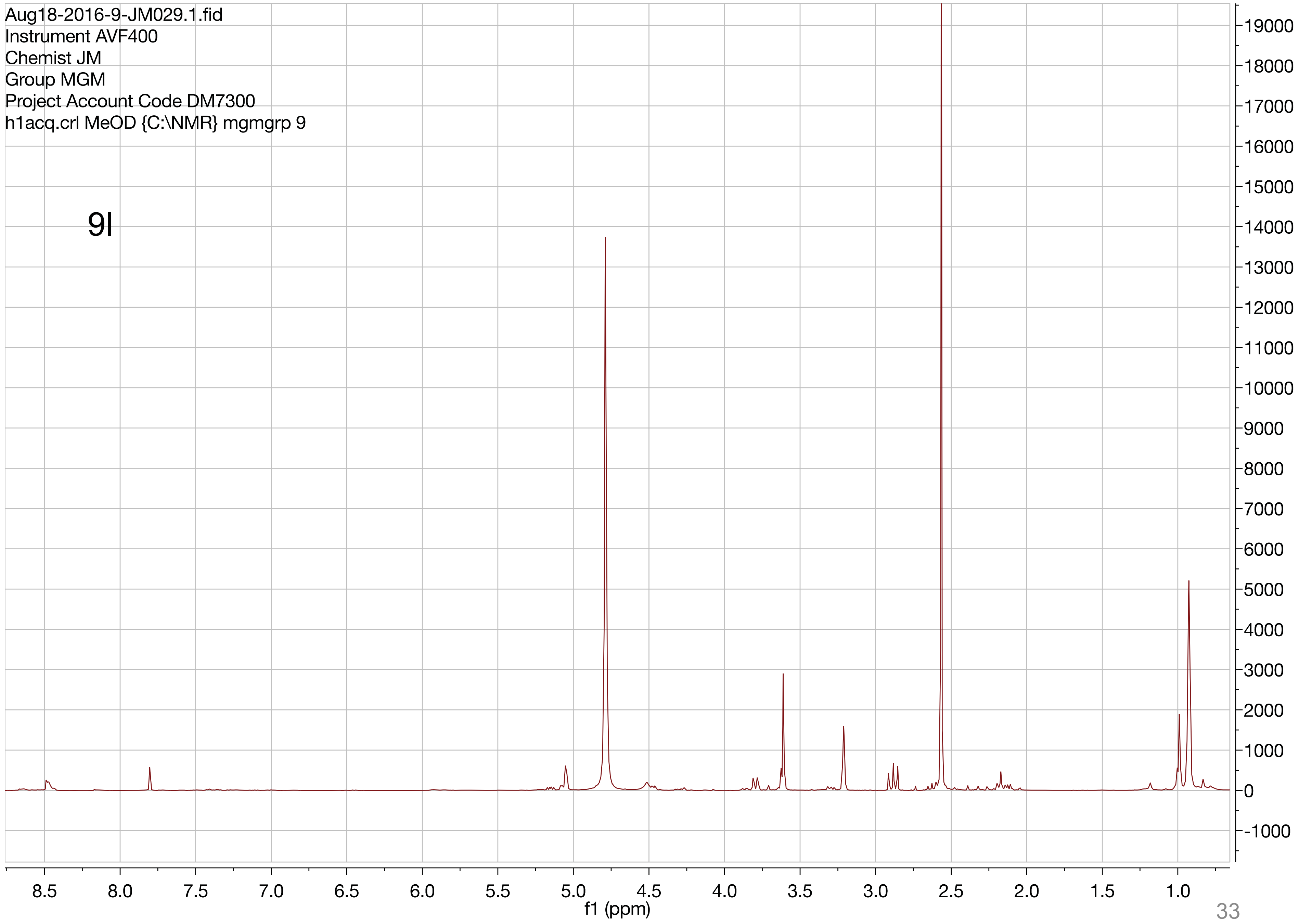
Aug18-2016-9-JM029.1.fid
Instrument AVF400
Chemist JM
Group MGM
Project Account Code DM7300
h1acq.crl MeOD {C:\NMR} mgmgrp 9
9l
f1 (ppm)

9l

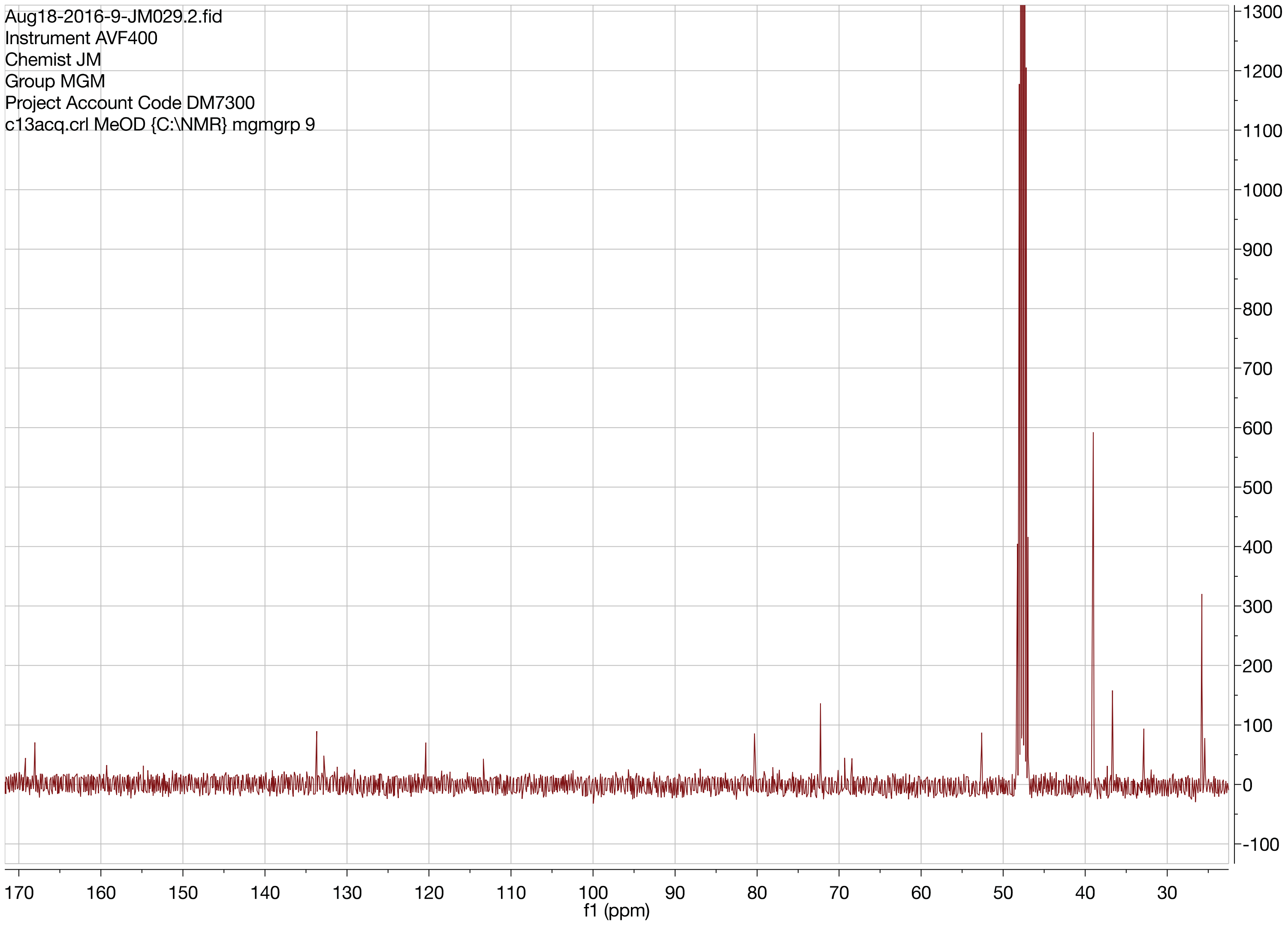
Aug18-2016-9-JM029.2.fid
Instrument AVF400
Chemist JM
Group MGM
Project Account Code DM7300
c13acq.crl MeOD {C:\NMR} mgmgrp 9
f1 (ppm)

9l

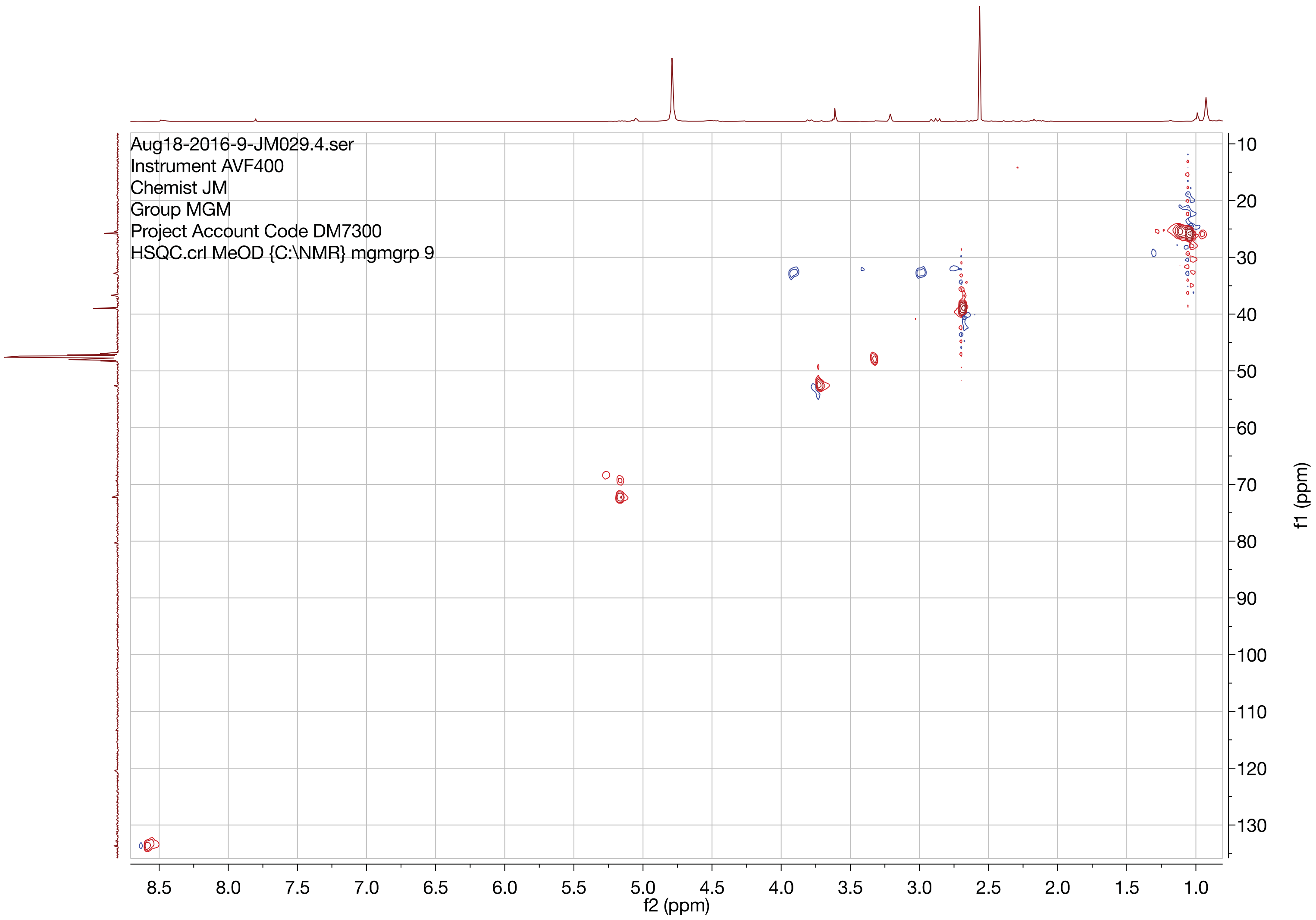
Aug18-2016-9-JM029.4.ser
Instrument AVF400
Chemist JM
Group MGM
Project Account Code DM7300
HSQC.crl MeOD {C:\NMR} mgmgrp 9
f2 (ppm)
f1 (ppm)
