# Supplementary material for: Fused-Ring Oxazolopyrrolopyridopyrimidine Systems with Gram-Negative Activity
Source: Antibiotics (Basel). 2017 Jan 13;6(1):2. doi: 10.3390/antibiotics6010002 (PMC5372982; doi:10.3390/antibiotics6010002)
Supplement: Supplementary file 1 [file antibiotics-06-00002-s001.zip › Supplementary Materials/Supplementary Figures and Tables.pdf]

antibiotics

MDPI

Supplementary Materials: Fused-Ring Oxazolopyrrolopyridopyrimidine Systems with Gram-Negative Activity

Yiyuan Chen, Jonathan G. Moloney, Kirsten E. Christensen and Mark G. Moloney

Table S1. Bioassary data for Ceph. C.

Bioassays Plate Calibration with Cephalosporin C (MW 415.4)						
Stock Solution µg/mL	Ceph. C µL/well	Water µL/well	Ceph. C µL/well	Ceph. C mmoles/well	Zone Size mm	Log (mmol/well)
For *Escherichia coli*						
10	20	80	0.2	0.481463649	n/a	−0.317
	40	60	0.4	0.962927299	n/a	−0.016
	60	40	0.6	1.444390948	14.5	0.160
	80	20	0.8	1.925854598	16.5	0.285
	100	0	1	2.407318247	21	0.382
For *Escherichia coli*						
100	20	80	2	4.814636495	36.5	0.683
	40	60	4	9.62927299	40	0.984
	60	40	6	14.44390948	40.5	1.160
	80	20	8	19.25854598	41.5	1.285
	100	0	10	24.07318247	42.5	1.382
For *Staphylococcus aureus*						
1000	20	80	20	48.1463495	n/a	1.683
	40	60	40	96.2927299	n/a	1.984
	60	40	60	144.4390948	n/a	2.160
	80	20	80	192.5854598	15.5	2.285
	100	0	100	240.7318247	17.5	2.382

n/a = not active.

Table S2. Bioassary data for solvents.

Solvent	Bioactivity
50% DMSO in MeOH	n/a
50% DMSO in H_2O	n/a
70% DMSO in H_2O	n/a
Pure DMSO	n/a

n/a = not active.

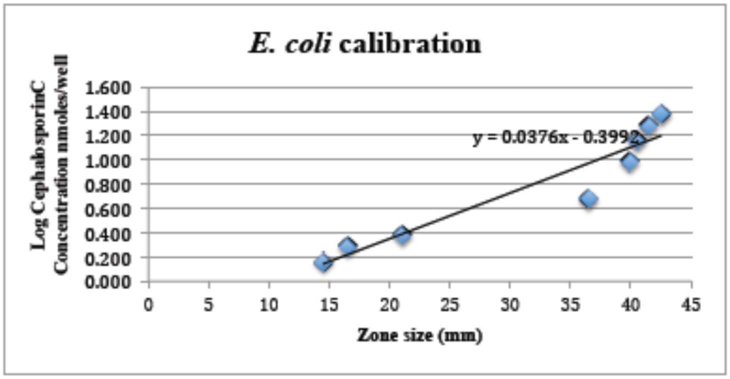

Figure S1. *E. coli* calibration.

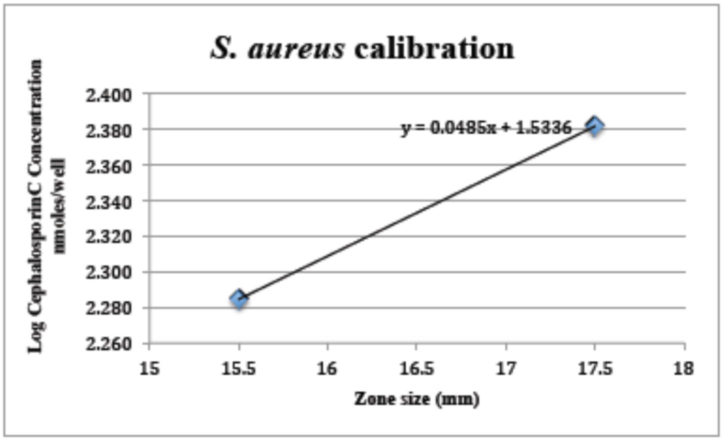

Figure S2. *S. aureus* calibration.

© 2017 by the authors; licensee MDPI, Basel, Switzerland. This article is an open access article distributed under the terms and conditions of the Creative Commons by Attribution (CC-BY) license (http://creativecommons.org/licenses/by/4.0/).
